# A new evaluation methodology study - Integrating ‘ancient literature - clinical research - expert consensus’ firstly proposes eight elements for taking Chinese medicine decoctions

**DOI:** 10.3389/fphar.2025.1585428

**Published:** 2025-08-08

**Authors:** Feiyu Li, Jing Shang, Yixuan Li, Guoxiu Liu, Sijin Zhao, Jiankun Wu, Hongmei Chen, Wanlin Wu, Xuelong Zhao, Huaqiang Zhai

**Affiliations:** ^1^ Standardization Research Center of Traditional Chinese Medicine Dispensing, School of Chinese Materia Medica, Beijing University of Chinese Medicine, Beijing, China; ^2^ Pharmacy Department, Beijing Hospital of Traditional Chinese Medicine, Beijing, China; ^3^ Pharmacy Department, Hangzhou Hospital of Traditional Chinese Medicine, Zhejiang, China; ^4^ French Confederation of Traditional Chinese Medicine (Confédération Française de Médecine Traditionnelle Chinoise, CFMTC), Paris, France; ^5^ Pharmacy Department, Nanjing Hospital of Traditional Chinese Medicine, Nanjing, China

**Keywords:** Chinese medicine decoctions, rational use of medicines, eight key elements for medication, delphi method, traditional Chinese medicine

## Abstract

**Background:**

The scientific and rational use of Chinese medicine decoctions can improve their compliance, safety and efficacy. However, there is a lack of national-level best practice guidelines on the management of Chinese medicine decoctions, particularly in the rational use of them. We aim to establish a comprehensive and standardised reference standard comprising key elements of Chinese medicine decoctions.

**Methods:**

This study was conducted using the method of “Ancient Literature - Clinical Research - Expert Consensus”. (1) Ancient literature analysis: Systematically analysed 1,019 records of Chinese medicine decoctions in *“Bijiqi Qianjin Yaofang”*, and initially extracted the elements related to the efficacy of the medicine. (2) Clinical research: A questionnaire covering the knowledge and demand of medication was designed and distributed to medical institutions in 12 regions, including Beijing and Shanghai, and 87 valid data were collected. The results showed that 69.62% of the practitioners had encountered difficulties in providing medication guidance, and 71.62% believed that standardised guidelines were urgently needed. Accordingly, a preliminary knowledge framework was formed. (3) Expert consensus: 20 Chinese and foreign clinical pharmacy experts (including France and U.S) were selected to prepare an advisory form based on the preliminary results, and a consensus was reached after two rounds of the Delphi method, confirming the content of the general and personalised guidance, covering terminology, usage, storage, contraindications and other dimensions, and finally identifying the eight core elements.

**Results:**

The method of “ancient literature-clinical research-expert consensus” is the first time to refine the eight elements of taking Chinese medicine decoctions. (1) Analysis of ancient literature reveals that temperature, course of treatment, frequency, dosage, time of administration, storage conditions, post-medication care and contraindications are the key influencing factors. (2) Clinical research confirmed the urgent need for standardised guidance, and a preliminary framework for a body of knowledge was constructed. (3) Standardised definitions of the eight elements were clarified through the Delphi Expert Consensus, including temperature, duration, frequency, dosage, time, storage conditions, post-medication care, and contraindications.

**Conclusion:**

This international guideline addresses a critical gap in taking Chinese medicine decoctions, offering an evidence-based framework for rational clinical use. Future efforts should prioritize expanded clinical validation and scenario-specific protocols to optimize standardization and safety.

## 1 Introduction

Medication guidance has been a major concern of the World Health Organization (WHO) and the International Pharmaceutical Federation (FIP) (International Pharmaceutical Federation, 2025). Researches have shown that providing scientific and rational medication guidance can effectively mitigate medication errors while facilitating patients’ acquisition of proper medication administration techniques, improve patients’ medication compliance, reduce the probability of adverse reactions, and ultimately ameliorate patients’ prognosis and quality of life (Rappaport et al., 2023; Yu, 2020; Duan, 2020). The history of patient medication guidance information services in Western countries is extensive (Zaky et al., 2021). During the dispensing process, pharmacists typically provide patients with reminders regarding drug precautions by incorporating supplementary materials such as pocket cards, drug labels, medication guides, consumer medication information, and patient information leaflets (Rappaport et al., 2023; Liang et al., 2022; Watterson, 2019).

The Chinese medicine decoctions, as one of the primary methods in Chinese medicine for treating diseases, offers numerous advantages including adjustability, rapid efficacy, easy absorption, low irritation, and minimal adverse reactions (Peng et al., 2024; Li et al., 2024). It has played an irreplaceable role in therapeutic interventions (Cho et al., 2024; Xin et al., 2020). The therapeutic process of Chinese medicine decoctions encompasses multiple sectors, including prescription, dispensing, decoction, and administration (Gui et al., 2013). Incorrect dosage, method, or treatment duration not only compromises drug efficacy but also poses potential risks of adverse reactions and even life-threatening consequences in serious cases (Li et al., 2018). As the final link before taking Chinese medicine decoctions, the efficacy of pharmacist’s medication guidance directly influences clinical outcomes (Qin and Zhang, 2021). Pharmacists inform patients and their families in detail about the usage, dosage, contraindications, precautions, possible adverse reactions and countermeasures related to Chinese medicine decoctions in simple, clear and easy-to-understand language, and guide patients to use Chinese medicine decoctions scientifically and correctly, which can solve the patients’ confusion about taking medication, guide patients to use medication reasonably, and promote the high-quality development of traditional Chinese medicine clinical pharmacy services.

As a crucial determinant influencing the level of rational drug use in clinical practice, authoritative foreign institutions and relevant professional societies have issued abundant drug guidelines along with their development manuals (Wilson, 2023). The principles for developing clinical practice guidelines have been successively established by reputable organizations such as the *World Health Organization* (WHO) (World Health Organization, 2024), *the National Institute for Health and Care Excellence* (NICE) (JinY et al., 2018), *the American College of Occupational and Environmental Medicine* (ACOEM) (American College of Occupational and Environmental Medicine, 2025) and so on. It is widely acknowledged that the formulation of clinical practice guidelines necessitates a consensus method to ensure scientific rigor. Among these methods, the most commonly used are the Delphi method (Jones and Hunter, 1995). However, due to the inherent subjectivity of Chinese medicine decoctions in diseases treatment, along with a lack of standards, methods, approaches, evaluation, and integration of traditional Chinese medicine literature and medical experience, as well as insufficient original research on methodology development for clinical practice guidelines in traditional Chinese medicine; China has not yet established a scientifically unified evidence quality grading system and experiences uneven guideline quality (Yu et al., 2023; Xie et al., 2024; Hatakeyama et al., 2023). Through a systematic analysis of 263 national, local, and industry standards issued over the past decade from 2015 to 2025, it was found that 80.6% (212/263) of the standards focused on the upstream industry of TCM (e.g., technical specifications for cultivation, production, and processing of TCM herbs), with a small number of standards regulating the process of dispensing of TCM (0.19%), and there was no standardised guidance on the taking of Chinese medicine decoctions. When searching international standards and standards of international organisations, the requirements for taking Chinese medicine decoctions are also vague, for example, there are no clear quantitative indicators for the concept of “warm administration” (World Federation of Chinese Medicine Societies, 2020). In addition, the research team conducted a survey on the necessity of setting up instructions for taking Chinese medicine decoctions for relevant practitioners in the early stage of the study, and 71.26% of the respondents thought that it was very necessary to set up such instructions, while 26.44% thought that it was necessary to set up such instructions. This all reveals the current lack of methodological level. Therefore, it is crucial to establish a set of guideline formulation methodology that adhere to international standards while being suitable for China’s national conditions and effectively reflect the characteristics and advantages of traditional Chinese medicine.

In this study, we conducted a comparative analysis of domestic and international guidelines on medication administration using data analysis methods. We identified the key information pertaining to the guidance on Chinese medicine decoctions. Additionally, we administered research questionnaires to hospital practitioners to find the crucial aspects and challenges associated with clinical utilization of Chinese medicine decoctions. Subsequently, we developed a comprehensive framework for the guidance of taking it based on these findings. Finally, employing the Delphi method, we carried out the expert consensus, and clarified the key factors that influence Chinese medicine decoctions intake.

## 2 Methods

### 2.1 Data analysis

The data analysis was divided into two parts. Firstly, the key elements required in the medication guidance were compared across four sources: the US medicines-taking guide, UK medication guidance labels, Australian consumer medication information leaflet, and the traditional Chinese medicine in Japanese drug inserts. This comparative analysis aimed to sort out the key points of medication guidance pertaining to Chinese medicine decoctions. Documents in Chinese, English, and Japanese were analyzed by native-speaking researchers fluent in both the source language. Each document underwent independent dual translation reconciliation. Secondly, the data were extracted from the “*Bei Ji Qian Jin Yao Fang*”, “*Shang Han Za Bing Lun*” and “*Ancient Catalogue of Classical Famous Prescriptions (the first batch)*” recorded in Chinese medicine decoctions. Subsequently, these data were entered into Microsoft Excel 2022 for analysis, followed by descriptive analyses of the data using SPSS 27.0 software.

Among them, the inclusion criteria for the decoctions were as follows: (1) the decoctions recorded in the aforementioned literature; (2) those who had a clear record of formula name, prescription composition, primary treatment diseases, temperature, the time, frequency, dosage, treatment course, contraindications, post-drug care and efficacy observation; (3) those who had a complete record of medicine dosage and frequency. Exclusion criteria: (1) Formulas in other dosage forms except Chinese medicine decoctions; (2) repeated recording of identical formulas.

### 2.2 Questionnaire survey

We employed an online questionnaires distribution method to analyze the differences between domestic and foreign medication guidance, integrating China’s Chinese medicine clinical pharmacy development. By summarizing the current situation and service requirements of Chinese medicine decoctions medication guidance, we adopted a Western approach to medication guidance developed mode while considering the distinctive characteristics of traditional Chinese medicine filed through research on integrated clinical practice. Guided by relevant state laws, regulations, and policy norms, this study aims to elucidate the *status quo* and demands for Chinese medicine decoctions medication guidance among hospital practitioners during their clinical work.

The questionnaire involves four types of questions: fill-in-the-blank, multiple-choice, scoring and sorting questions. It divided into three parts: basic information (8 questions), knowledge of guidance on taking Chinese medicine decoctions (18 questions), and demand for guidance on taking Chinese medicine decoctions (6 questions). Among them, the basic information includes six items regarding the participants working area, nature and type of working unit, position, years of working experience, and the highest education level. The section on knowledge about the guidance of taking medication in Chinese medicine decoctions includes seven items related to frequency, person in charge of medication guidance, form and experience of guidance provision, effectiveness, and basis for the guidance. Lastly, the section on demand for guidance broths covers the problems encountered by participants as well as their perceived necessity and need for such guidance.

### 2.3 Delphi survey

Following the principles of the Delphi method, we adopted a systematic and sequential approach characterized by participants anonymity. Twenty experienced experts from Beijing and Hangzhou, specializing in clinical practice, pharmacy services, management, education, etc., were selected. The principles for expert selection are as follows: (1) Selection of experts specialized in clinical Chinese medicine and pharmacy services, who possess a high level of proficiency in this field, have established visibility, hold senior titles with extensive clinical experience, and demonstrate interest and perseverance in completing guideline development tasks; (2) Clinical experts including chief pharmacists and members from relevant professional societies, considering representation from various hospitals and geographical locations; (3) Adherence to the principles of representativeness, extensiveness and authority when selecting experts while also considering the purpose and subject of the study by incorporating relevant professional fields and geographical distribution; (4) Consideration of clinical pharmacists who have been actively involved in clinical Chinese medicine-related work for more than 10 years with an intermediate or higher title.

**FIGURE 1:**
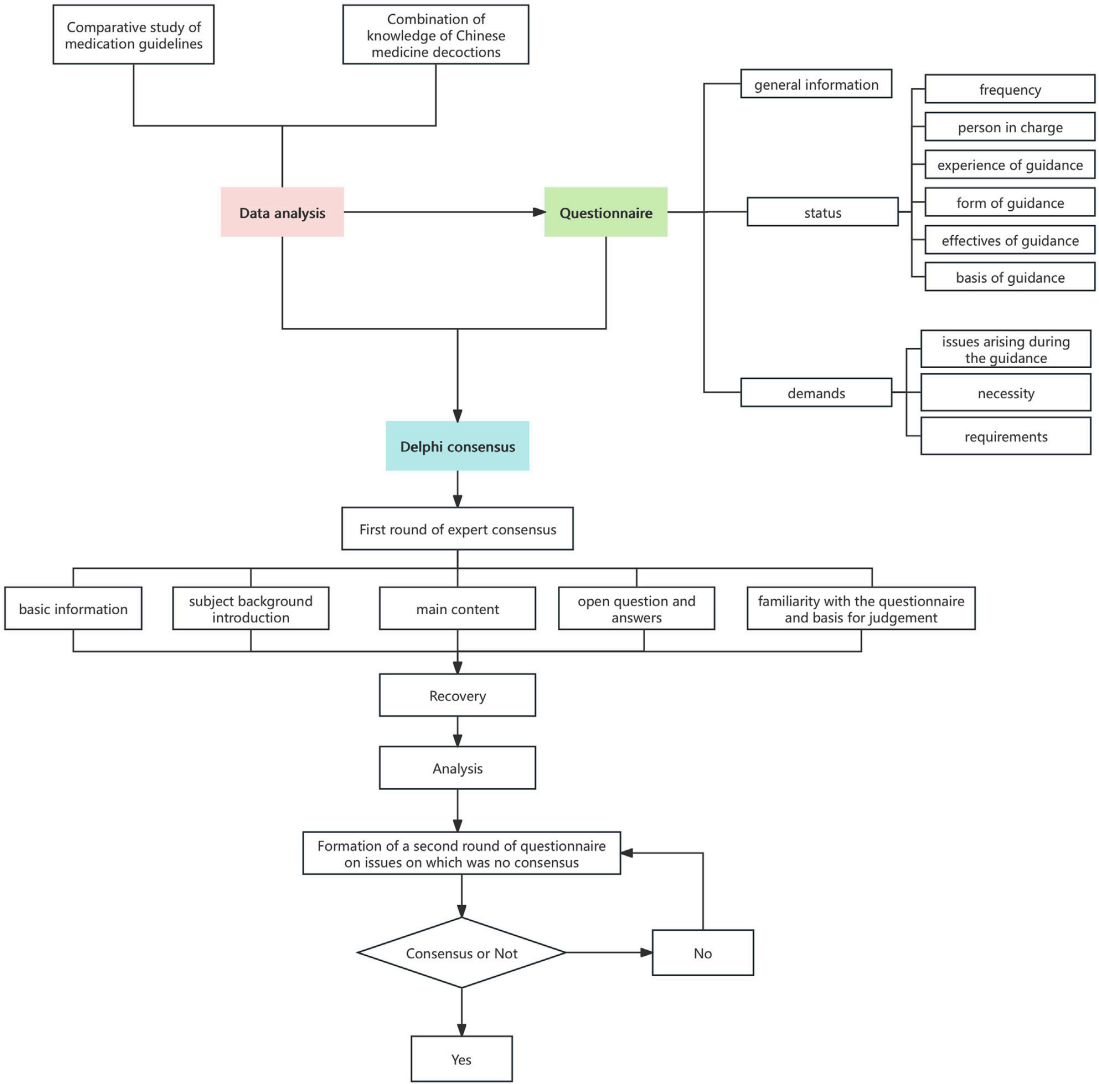
Screening process for key elements of the medication guidelines.

The correspondence questionnaire is structured into five parts: basic information, subject background introduction, main content of the questionnaire, open questions and answers, and experts’ familiarity with the questionnaire and the basis for judgment. Within the main content section, relevant indices, and the weighting of the guidance content on the administration of Chinese medicine decoctions are presented to experts who are asked to rate both importance and satisfaction levels. Based on these ratings, values are assigned to each index according to its significance. After collecting the questionnaires, the experts’ opinions were identified and screened based on statistical analysis indicators. Subsequently, a comprehensive evaluation was conducted by considering the results of internal group deliberation to exclude, incorporate or revise these expert opinions. For the elements that experts failed to reach a consensus, a second round of expert questionnaires was conducted. In this round, the mean value, coefficient of variation, and full score ratio from the first round were provided for each element. The experts were then asked to re-evaluate the adjusted structural framework and derive results. In summary, the eight elements screening process for taking Chinese medicine decoctions guidelines is shown in Figure 1.

### 2.4 Consensus definitions

For the Delphi survey administered to professionals, the consensus definitions were: (i) The importance of the elements was assigned according to a 5-point “Likert scale”, and the scoring results were expressed according to the arithmetic mean ( $\bar{X}$ ), perfect score ratio (K), sum of ranks (Si), weighting coefficient (Wi), and standard deviation (SD); it was considered that $\bar{X}$ >3.50, K > 0.20, and Si > 35 indicated a high degree of concentration of experts’ opinions. (ii) The coefficient of variation Cv is used to indicate the degree of coordination of experts’ opinions; the smaller the coefficient of variation, the higher the necessity of coordination of experts’ opinions on the indicators, and the general Cv < 0.25.

## 3 Results

### 3.1 Screening of core elements of guidance for taking Chinese medicine decoction

#### 3.1.1 Comparative study of the content of medication guidelines

The International Pharmaceutical Federation (FIP) has issued *Guidelines for the labels of prescribed medicines* (The International Pharmaceutical Federation, 2001), recommending that the medication labels should include both general information and patient’s personalized information, employing easily comprehensible language to facilitate accurate preparation, administration, and storage of medicines. This approach aims to ensure optimal therapeutic effect while minimizing the occurrence of medication errors. Cautionary and advisory labels for dispensed medicines are attached to the appendix section of the *British National Formulary* (British Medical Association and the Royal Pharmaceutical Society, 2009), which contains two parts: an attention-to-use label and a dosage and route of administration label. This section is further divided into 30 sub-labels, enabling pharmacist to provide additional explanations regarding precautionary measures during medicine dispensation. By comparing the medication guidelines (U. S. Food and Drug Administration, 2023) published by the US Food and Drug Administration, the Australian consumer information leaflet (Australian Government Department of Health and Aged Care, 2020), and the instructions for taking Japanese Han prescription medicine (Tsumura and Co, 2015), it was found that a higher emphasis was placed on the instructions, dosage, side effects, adverse reactions and storage conditions of the medicines, given in Table 1.

**TABLE 1:** Comparative study of the content of medication instructions in different countries.

Coverage	FDA medication guide	Australian consumer information leaflet	Instructions for taking Japanese han prescription medicine
The most important information about the Drug-X	✓	—	—
Drug description	✓	✓	✓
Who should not take Drug-X	✓	—	✓
All the medical conditions	✓	✓	—
How to take the medicine	✓	✓	✓
Warnings and precautions	—	✓	
Avoid during medication	✓	—	✓
Possible side effects	✓	✓	✓
Potential interactions between the medication and food or other medications	—	✓	—
Management strategies for drug overdose incidents	—	✓	—
Storage condition	✓	✓	✓
General information	✓	—	✓
Ingredients	✓	✓	—
Signature of physician or pharmacist	—	—	✓
Name and address of the sponsor	—	✓	—
Date the CMI was last updated	—	✓	—

#### 3.1.2 The content of the knowledge combined with Chinese medicine decoctions

The therapeutic efficacy of Chinese medicine decoctions is influenced by various factors, encompassing eight key elements: temperature, duration, frequency, dosage, treatment course, contraindications, post-drug care, and therapeutic effect performance. The regular temperature is thermally neutral, closely resembling the human body’s natural environment; in addition, it is hot when treating cold symptoms and cold when treating hot symptoms.

The time should be suited to the changes in the condition and the body’s rhythms. Medications that cause irritation to the stomach and intestines, drugs with higher toxicity levels, digestive drugs, diuretics, and drugs for treating dizziness, headache, sore throat, heart conditions, lung disorders, chest ailments, diaphragm issues and other epiglottis-related conditions should be taken after meals to minimize gastric and intestinal irritation. Medications that have toning effects on the body or act as, diuretics or strengtheners for the spleen and stomach; those used for treating liver and kidney deficiencies; intestinal disorders, general malaise, lumbago; as well as other disorders below the chest and diaphragm should be taken before meals to optimize their impact on the stomach lining. Cathartic medications including laxatives or purgatives intended for relieving constipation or treating disease of limbs and blood vessels should be taken on an empty stomach to avoid any influence from food. Purgative medications specifically designed for water-expelling diseases are recommended to be taken at the beginning of each morning. Medications prescribed for malaria treatment should be taken during periods of attack. Medications used in acute or critical illnesses management should be taken promptly upon onset.

The recommended frequency for treatment of acute and serious diseases, abortifacient type, gastrointestinal diseases, and lower body aliments is 2–3 times per day. For toxic and intense decoctions, the recommended frequency is once daily, while for tonic decoctions it is four times daily. In cases of acute and serious diseases such as fever, vomiting, diarrhea, etc., frequent administration may be necessary. The dosage is closely related to the frequency of administration with a common dose being 200 mL which can serve as a reference for the specification of single packet dosage of Chinese medicine substitute decoction. However, in situations where there are fewer prescribed medicinal flavors, the patient is weak, or when treating phlegm, edema and other aspects of the disease, smaller dosages maybe appropriate whereas larger dosages can be considered for Lower Jiao diseases.

The duration of Chinese medicine decoctions treatment is related to the change of the condition, with shorter courses typically prescribed for superficial evidence, mild ailments, and external diseases, while longer courses are recommended for severe disease, deficiency evidence, and chronic diseases. Besides, in cases where patients exhibit acute symptoms such as hematuria or diarrhea accompanied by pain or vomiting, treatment should be discontinued once the disease shows signs of improvement.

The contraindications of Chinese medicine decoctions can be categorized into drug, disease, food, and environmental contraindications. Raw and cold products possess a cooling nature that may impair the spleen and stomach’s yang qi. Greasy foods, characterized by their high fat content and thick consistency, are difficult to digest and can generate internal heat, thereby exacerbating the burden on the gastrointestinal system. Warm-nature meats such as dog meat and mutton tend to promote excessive bodily heat. Spicy or stimulating foods with pungent flavors and dry heating properties tend to induce wind movement in the body, which can worsen existing conditions. Therefore, it is advisable to avoid consuming these items during medication.

Post-medication care of Chinese medicine decoctions encompasses dietary care and lifestyle interventions to enhance therapeutic efficacy. Dietary measures involve sipping porridge, drinking hot water, or incorporating other medicinal diets alongside medication intake, while lifestyle care mainly includes sweating, thermal insulation, and warming the bed to regulate qi circulation and optimize treatment outcomes.

The assessment of treatment efficacy plays a crucial role in predicting disease recurrence, determining resolution of the condition, timely cessation of medication, prognostication, and preemptively addressing potential doctor-patient disputes. Among these factors, excreta observation primarily involves monitoring sweat production, vomiting frequency, stool consistency and volume, as well as the presence of blood. These indicators help determine disease progression while considering subjective patient feedback to guide treatment duration and drug suitability.

### 3.2 Analysis of questionnaire survey results

**FIGURE 2:**
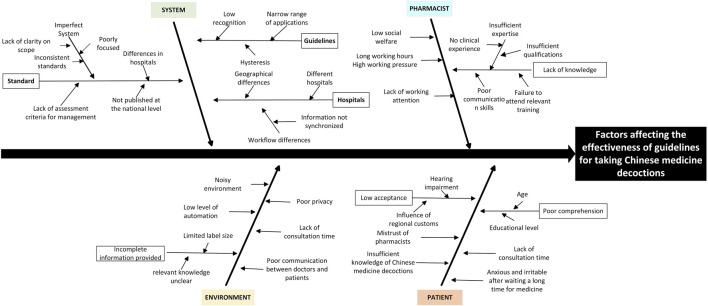
Factors affecting the effectiveness of guidelines for taking Chinese medicine decoctions.

Stratified sampling method was used to select the medical institution practitioners in 13 provinces and cities of Beijing, Shanghai and Zhejiang Province in China from 20 June 2023 to 30 June 2023, and a total of 90 questionnaires were distributed, of which, the time of answering the questionnaire was much less than the average time, or the number of unanswered questions was more than 2/3 of the total number of questions, or all the answers were the same answer was judged as invalid questionnaires. After screening, 87 valid questionnaires were recovered, with an effective recovery rate of 96.67%. The characteristics of the questionnaire research participants are shown in Table 2. The questionnaire content is shown in Supplement 1. The results of the research showed that there are still many irregularities in the current guidance for taking Chinese medicine decoctions, which will affect the clinical efficacy of Chinese medicine decoctions in different degrees. The existing problems can be analyzed from four perspectives: environment, system, pharmacist and patient, as shown in Figure 2.

**TABLE 2:** The characteristics of participants in the questionnaire survey.

Characteristics	No.of population
Type of work unit	
Chinese medicine hospital	62 (62/87, 71%)
General hospital	17 (17/87, 20%)
Western medicine hospital	6 (6/87, 7%)
Others	2 (2/87, 2%)
Specialty	
Physician	8 (8/87, 9%)
Traditional Chinese medicine pharmacist	72 (72/87, 83%)
Western pharmacist	4 (4/87, 5%)
Nurse	2 (2/87, 2%)
Manager	1 (1/87, 1%)
Years of experience	
5 years or less	9 (9/87, 10%)
6∼10 years	17 (17/87, 20%)
11∼20 years	39 (39/87, 45%)
More than 21 years	22 (22/87, 25%)
Education background	
Doctor	6 (6/87, 7%)
Master	21 (21/87, 24%)
Undergraduate	60 (60/87, 69%)

#### 3.2.1 Noisy environment and low level of automation in Chinese medicine pharmacy

According to the results, 35.63% of the respondents indicated that they would combine verbal instructions and paper instruction sheets in the process of medication instruction, while 54.02% of the respondents indicated that the pharmacist would only give verbal instructions to the patients when dispensing medication. Due to the limited space and inadequate infrastructure of the Chinese medicine pharmacies, patients often experience long waiting time and the narrow space, which can lead to increase irritability and dissatisfaction. Additionally, the noisy environment in these pharmacies, coupled with various factors such as differences in patients’ personalities and values, may contribute to some patients being reluctant to engage in detailed medication counseling or struggling to remember the pharmacist’s instructions within a relatively short period. Therefore, patients may still experience confusion regarding the administration of Chinese medicine decoctions despite receiving verbal instructions alone.

#### 3.2.2 Low standardization of medication instruction for Chinese medicine decoctions

It was found that 69.62% of the researchers encountered challenges during the medication instruction process, with 63.64% attributing these difficulties to the absence of well-defined standards for medication instruction. Currently, there is a lack of national legislation, regulations, and implementation guidelines regarding medication guidance for Chinese medicine decoctions. As a result, hospitals at different levels operate based on their own requirements, leading to noticeable geographical and institutional vitiations. Additionally, the absence of performance assessment standards for medication guidance staff hinders the ability to quantitatively evaluate patient satisfaction with the service, potentially diminishing staff motivation and effectiveness. The lack of standardized performance appraisal criteria for medication counseling staff may significantly diminish staff motivation and work efficacy.

#### 3.2.3 Awareness of pharmacy services among physicians and pharmacists needs to be enhanced

According to the results, during the process of clinical practice, the main reference basis for the guidance of Chinese medicine decoctions is clinical experience. However, there is a need for improvement in terms of professional and systematic training, as well as standardization, comprehensiveness, and accuracy in practical guidance. Furthermore, current clinical practice still require enhancements in terms of guidelines for using decoctions to treat related diseases. The expert consensus lags to some extent and there is room for improvement regarding the breadth and depth of authoritative reference information.

#### 3.2.4 Lack of patients’ knowledge about Chinese medicine decoctions

The administration of Chinese medicine decoctions is a complex process influenced by factors such as patients’ education level, age, and cognitive abilities, etc. The level of adherence to medication administration instructions varies, for instance, elderly patients may experience challenges comprehending or retaining the pharmacist’s guidance during the medication intake process. The poor taste of decoctions, coupled with factors such as forgetfulness, busyness, contributes to patients’ low adherence. This often leads to arbitrary discontinuation or omission of medication, thereby compromising the clinical efficacy of Chinese medicine decoctions. In addition, influenced by the concept of “valuing medicine over medicine”, certain patients exhibit limited acceptance and trust in the medication guidance provided by pharmacists, thereby augmenting the challenges associated with implementing medication counseling efforts.

### 3.3 Key elements affecting the administration of Chinese medicine decoctions

In accordance with the principles of authority, representativeness, and comprehensiveness in expert selection within the Delphi method, and considering both regional and disciplinary representation, a total of 20 highly experienced experts specializing in clinic practice, pharmacy services, management, and education from Beijing and Hangzhou were selected for this study, the characteristics of participants in the Delphi survey are shown in Table 3. The data were processed using Excel 2022 and SPSS 27.0 software. Qualitative data were described as a percentage of the maximum score (%), while quantitative data were described using measures such as mean, standard deviation, and coefficient of variation. Additionally, the reliability of the study results was evaluated based on experts’ consensus rating in terms of positivity, authority, opinion concentration, and coordination.

**TABLE 3:** The characteristics of participants in the Delphi survey.

Characteristics	No. of population
Gender	
Male	3 (3/20, 15)
Female	17 (17/20, 85%)
Age	
30-39	4 (4/20, 20%)
40-49	7 (7/20, 35%)
50-59	9 (9/20, 45%)
Education background	
Doctor	2 (2/20, 10%)
Master	6 (6/20, 30%)
Undergraduate	12 (12/20,60%)
Years of experience	
10 years or less	1 (1/20, 5%)
11–15 years	4 (4/20, 20%)
16–20 years	3 (3/20, 15%)
More than 20 years	12 (12/20, 60%)
Type of work unit	
Chinese medicine hospital	17 (17/20, 85%)
General hospital	3 (3/20, 15%)
Field of expertise	
Chinese Medicine Clinical Pharmacy	9 (9/20, 45%)
Traditional Chinese Medicine, Chinese Medicine Preparation	6 (6/20, 30%)
Pharmacy management, pharmacy services	4 (4/20,20%)
Chinese herbal medicine concocting	1 (1/20, 5%)

#### 3.3.1 Round 1 of the Delphi survey

The first round of Delphi questionnaires was conducted from July 17 to 24 July 2023. The recovery rate achieved in this study is 100%, with a familiarity coefficient (Cs) of 0.680 and an expert authority coefficient (Ca) of 0.954, indicating that the judgment based on the coefficient (Cr) is 0.817 > 0.7. Therefore, it can be concluded that the experts selected for this study possess a high level of expertise. The experts’ average scores on the indicators all exceed 3.5, with the sum of scores in the first-round surpassing 23. These results indicate a high level of consensus among experts regarding the necessity of elements at all levels. The coefficient of concordance among experts’ ratings on the necessity of elements at all levels is 0.251 (χ^2^ = 314.053, P = 0.000 < 0.05). Additionally, the coefficients of variation (Cv) for experts’ ratings on the necessity of knowledge entries at all levels are less than 0.25, indicating a high level of agreement among experts regarding these issues. Through the application of hierarchical analysis method, it is evident that experts deem the logic behind the guidance framework for Chinese medicine decoctionss to be reasonably constructed, with high acceptance of consistency in the guidance model demonstrated in Table 4 and 5.

**TABLE 4:** Results of the evaluation of the hierarchical analysis of the second level elements.

Elements	Item name	Eigenvector	Weight value	Maxi-mum eigen-value	CI value	RI value	CR value	Consistency test results
General guidance	Name of the decoction and its basic effects	2.008	66.0943%	3.015	0.007	0.520	0.014	Pass
	How to take your medicine	0.760	25.339%					
	Storage condition	0.232	7.718%					
Personali-sed Guidance	Warnings	2.348	58.693%	4.160	0.053	0.890	0.060	Pass
	Contraindications	1.065	26.635%					
	Aftercare	0.324	8.091%					
	Contact Pharmacist	0.263	6.582%					

**TABLE 5:** Evaluation results of the three-level hierarchical analysis of elements.

Elements	Item name	Eigenvector	Weight value	Maxi-mum eigen-value	CI value	RI value	CR value	Consistency test results
Methods for medication administration	Temperature	0.801	16.027%	5.407	0.102	1.120	0.091	Pass
	Time	1.838	36.764%					
	Frequency	1.097	21.937%					
	Dosage	0.637	12.739%					
	Treatment Course	0.627	12.534%					
Contraindications	Drug-Drug	2.410	48.205%	5.337	0.084	1.120	0.075	Pass
	Drug-Population	0.853	17.509%					
	Drug-Food/Health Products	0.748	14.961%					
	Drug-Disease	0.782	15.644%					
	Drug-Environment	0.204	4.132%					
Aftercare	Post-drug care	1.896	37.915%	5.148	0.037	1.120	0.033	Pass
	Adverse reactions and treatment	1.261	25.219%					
	Missed dose/overdose treatment	0.990	19.807%					
	Effect on excretion	0.322	6.434%					
	Self-monitoring	0.531	10.625%					

More than 10 participants provided significant proposals for round 1 of the Delphi survey. After discussion with the steering group, three indicators were added: (1) “Drug-population” was added under Contraindications for taking Chinese medicine decoctions. (2) A reference guideline was added under Contraindications for drug-drugs. (3) A reference guideline was added under Post-drug care. Deletion of four indicators: (1) Deletion of the item Contact Pharmacist. (2) Deletion of a reference guideline under Medication Administration Temperature. (3) Deletion of a reference guideline under Medication Administration Time. (4) Deletion of a reference guideline under Storage Conditions. Indicators that have been modified or adjusted will be marked during the round 2 of the Delphi questionnaire process.

#### 3.3.2 Round 2 of the Delphi survey

The second round of Delphi questionnaires was conducted from August 20 to 28 August 2023. Online distribution in the form of e-mail, based on the distribution of the first round of questionnaires, the structural framework of the elements of the guidance for taking Chinese medicine decoctions was modified, and the mean value, coefficient of variation, and full score ratio of the first round of experts’ ratings of the elements were also given, the main purpose of which was to ask experts to re-score the adjusted structural framework and to obtain the final results.

The return rate of the second round of the experts’ correspondence questionnaire was 100%, which indicated that the experts were more positive about the research. Through the experts’ reanalysis of the modified indicators, it was found that the mean value of the second round of expert scores were all >0.35, the full score ratio was >0.20, the coefficient of variation was <0.25, and the rank sum was all >35. Finally, after two rounds of correspondence questionnaire, the expert consensus was established.

**FIGURE 3:**
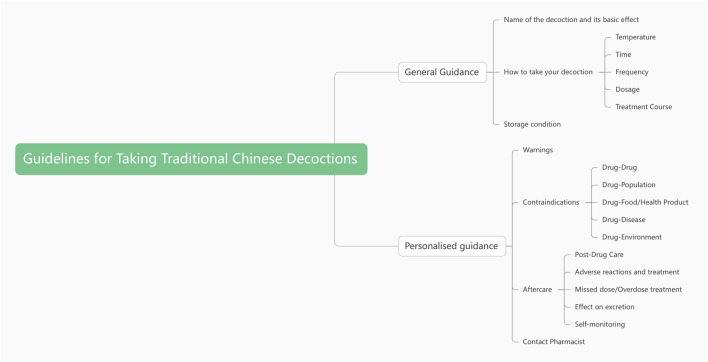
Key elements affecting the efficacy of taking Chinese medicine decoctions.

Consequently, the key factors affecting the efficacy of taking Chinese medicine decoctions were finally identified as two first-level elements: general guidance and personalized guidance. Additionally, eight second-level elements were recognized: temperature for administration, time of taking medicine, frequency, dosage, duration, storage conditions, post-drug care and contraindications to the taking of medicine, as shown in Figure 3.

## 4 Discussion

In recent years, with the rapid development of the discipline of traditional Chinese medicine, Chinese medicine decoctions have gained significant recognition and application across various clinical domains. However, with the gradual and extensive application of decoctions, some physicians excessively rely on modern equipment during the diagnosis and treatment process, thus neglecting the identification and treatment of traditional Chinese medicine (TCM). Consequently, they rigidly adhere to Western medicine’s perspective when using decoctions, lacking the characteristics of TCM and subsequently affecting their clinical efficacy. Moreover, various factors such as freshly preparing most decoctions at the time of clinical use, the complexity of the decocting process, inconvenience in portability, and poor taste further necessitate improvements in patients’ acceptance of and compliance with Chinese medicine decoctions. Therefore, it is of great significance to establish a medication guide for the administration of decoctions to standardize the method of taking decoctions, enhance patients’ adherence to medication, and ensure the clinical efficacy of decoctions.

In this study, by comparing the medication guide and drug instruction labeling in different countries, we found that European and American countries have a long history of information services for patient medication guidance, and their medication guide or drug instruction labeling has simple and easy-to-read language that is suitable for the reading level of the general public. In terms of content, all of them contain basic information of drugs, precautions, contraindications, and reaction descriptions, in which the contraindications and adverse reaction information of drugs for special groups are emphasized to a higher degree, which provides a reference for the guidance of Chinese medicine decoction taking. In contrast, there are no relevant instructions for Chinese medicine decoctions in China, and the records of adverse reactions and precautions are relatively scattered, which can be used to construct the guidance for taking Chinese medicine decoctions by reference to the instructions. At the same time, as a unique medical resource in China, Chinese medicine decoctions should be combined with ancient classic books and medication experience to give full play to its advantages. It was finally determined that the medication guide for Chinese medicine decoctions contains basic efficacy, medication method (including medication time, medication temperature, number of times of medication, dosage, and medication course), storage conditions, medication contraindications (including medication-drug contraindications/precautions, medication-population contraindications/precautions, medication-disease contraindications/precautions, medication-food/healthcare contraindications/precautions and medication-environmental contraindications/precautions), and post-dose care. precautions) and 5 parts of post-dose care (including appropriate post-dose care, possible adverse reactions and preventive/rescue measures, measures to deal with omission/overdose, effects on excretion, and how to conduct medication monitoring/self-recording). Combined with the results of the previous literature research, the research questionnaire was designed with the aim of understanding the pain points and difficulties in the clinical use of Chinese medicine decoctions medication guidance, clarifying the content and scope of Chinese medicine decoctions medication guidance, improving the knowledge system of Chinese medicine decoctions medication guidance, and providing references to the standardization of the construction of Chinese medicine decoctions medication guidance. The results of the research show that due to the low degree of automation of traditional Chinese medicine pharmacy, the lack of national standards, the low degree of standardization of medication instruction for traditional Chinese medicine decoctions, the lack of information awareness of medication instruction and pharmacy service among medical personnel, and the lack of patient’s knowledge of Chinese medicine decoctions, there are still many irregularities in the current medication instruction for Chinese medicine decoctions, which may affect the clinical efficacy of Chinese medicine decoctions to different degrees, and it is necessary to standardize the content of the medication instruction for Chinese medicine decoctions. There is an urgent need to standardize the content of Chinese medicine decoctions medication instruction. Therefore, combining the results of the previous literature analysis and questionnaire research, we compiled an expert correspondence questionnaire and adopted the Delphi expert correspondence method to construct the framework of the knowledge base of the guidance of taking Chinese medicine decoctions, and then revised and improved it according to the feedback from experts, fully combining the ancient guiding ideology of our country and the practical clinical work experience of experts, to summarize the key contents and relevant indexes of the knowledge base of the guidance of taking Chinese medicine decoctions. The results of the first round of expert consultation added drug-population contraindications, and added two monographs as reference guidelines, to better ensure the safety of medication, and to improve the science and accuracy of medication ordering service. In addition, the reference guideline of “Reference to Clinical Randomized Trials” under the item of temperature and time of drug administration was deleted, considering that the reference scope of the guideline was too wide and the quality of clinical reference was inconsistent; considering that the Chinese Pharmacopoeia only applies to China and does not represent the requirements of drug regulatory documents of overseas countries, the reference to the Chinese Pharmacopoeia under storage conditions of drugs was deleted; considering that “contact pharmacist” is too redundant and not universally applicable, the item was deleted. After the second round of expert consultation, the revised framework of elements reached a consensus and confirmed the eight elements for taking Chinese medicine decoctions, which including Taking Time, Frequency of Dosage, Taking Temperature, Dosage, Course of Treatment, Storage Guidelines, Special Taking Method, and Medication Self-Recording.

Based on these key factors, we have developed the international organization standard ‘*Guidelines for Taking Commonly-Used Chinese Medicinal Decoctions*’ (SCM 74–2024), and it has been published in March 2024. The guideline provides guidance on taking commonly used Chinese medicine decoctions, and quantifies the time and temperature of taking different diseases (e.g., the temperature for “warm” is 37°C–40°C). At the same time, personalised instructions for taking the decoctions are also provided for special groups such as pregnant women, the elderly, and children, such as in the “Dosage” entry, which stipulates the dosage for people of different ages: for adults, each dose should be 200–300mL; for infants less than 1 year old, each dose should be 1–5 mL (the liquid should be concentrated); for children 1–2 years old, each dose should be 20–40 mL (the liquid should be concentrated); for preschoolers 3–7 years old, each dose should be 40–80mL; for children 8–16 years old, each dose should be 100–150 mL. In the entry for contraindications: drug-population contraindications, it is indicated that the Chinese medicine herbs is contraindicated for women during pregnancy and the existence of contraindications between the elderly and children. In special cases or as directed by the physician. It can provide a reference basis for clinical pharmacists in the guidance of dispensing Chinese medicine decoctions. Next, we will carry out medical practice in medical institutions, decoction centers and other relevant places, and at the same time, relying on the Research Centre of traditional Chinese Medicine Dispensing, Beijing University of Chinese Medicine to promote the standard, relying on the academic meetings of the World Federation of Chinese medicine Societies Chinese Medicine Dispensary Professional Committee to promote the standard, and to provide practitioners of Chinese medicine with guiding criteria for the taking of traditional Chinese medicinal decoctions.

## 5 Limitation

Although this study constructed a more systematic system of elements of guidance for taking Chinese medicine decoctions, there are still certain limitations in the scope of the study and the representativeness of the data. On the one hand, the number of experts included in the expert consensus stage was relatively limited and dominated by Chinese regional experts, which may have affected the breadth of the international perspective to some extent. On the other hand, the questionnaire survey mainly covered Chinese medicine hospitals and combined Chinese and Western medicine hospitals in China, and although the sample size is representative, it has not yet fully covered all types of medical institutions in the country, which may have certain limitations in revealing the full picture of the current situation of the use of Chinese medicine decoctions. In addition, as the Delphi expert correspondence method was used in this study, the results of the judgement were mostly dependent on the expert’s own field of knowledge and clinical experience, which lacked reproducibility to a certain extent. Therefore, the follow-up study can further expand the source of experts and the scope of the survey to enhance the applicability and universality of the research results in different regions and clinical scenarios, and to improve the practical guidance value of the knowledge system.

## Data Availability

The original contributions presented in the study are included in the article/Supplementary Material, further inquiries can be directed to the corresponding authors.
