# Supplementary material for: A new evaluation methodology study - Integrating ‘ancient literature - clinical research - expert consensus’ firstly proposes eight elements for taking Chinese medicine decoctions
Source: Front Pharmacol. 2025 Aug 8;16:1585428. doi: 10.3389/fphar.2025.1585428 (PMC12371335; doi:10.3389/fphar.2025.1585428)
Supplement: Supplementary file 1 [file Supplementaryfile1.pdf]

ICS 11.020

SCM

世界中医药学会联合会
World Federation of Chinese Medicine Societies

SCM 74-2024

常用中药汤剂服用指南
Guidelines for taking commonly-used Chinese medicine decoctions

世界中联国际组织标准
International Standard of WFCMS

2024-03-14 发布实施
Issued & implemented on 14, Mar., 2024

目　次

前　言 I
引　言 II
1 范围 3
2 规范性引用文件 3
3 术语和定义 3
4 中药汤剂服用基本原则 3
5 中药汤剂服用通用性指导 4
6 中药汤剂服用个性化指导 7
附录 A（规范性） 10
附录 B（资料性） 11
附录 C（资料性） 15
参考文献 32

前　言

本文件的某些内容可能涉及相关专利，本文件的发布机构不承担识别专利的责任。

主要起草单位：北京中医药大学、杭州市中医院、中国-法国中医药中心（CFCMC）、首都医科大学附属北京中医医院、浙江省人民医院。

参与起草单位：北京市中西医结合医院、杭州唐古信息科技有限公司、法国中医联盟（CFMTC)、法国 KNP 集团、美国大有集团、日本明本株式会社、香港中医医院发展计划办事处、中国科学院中关村医院、北京中医药学会、中国中医科学院西苑医院、北京中医药大学第三附属医院、浙江省中医院、浙江省中西医结合医院、上海市第六人民医院、成都中医药大学附属医院、湖北省中医院、南京市中医院、甘肃省中医院、上海市中医医院、山东省中医院、广东省中医院、上海中医药大学附属岳阳中西医结合医院、北京大学第三医院、南方医科大学中西医结合医院、上海市浦东新区光明中医医院、青岛市中医医院、沧州市中西医结合医院、北京卫生职业学院、浙江中医药大学中药饮片有限公司、北京东华原医疗设备有限责任公司、北京和利康源医疗科技有限公司、新奇康药业股份有限公司、北京科园信海医药经营有限公司、上海同济堂药业有限公司、国药集团北京华邈药业有限公司。

指导委员会专家：王永炎、金世元、乔延江、王燕平、张华敏。

主要起草人：翟华强、陈红梅、吴宛霖（法国）、吴剑坤、张国兵。

参与起草人：

中　　国：韩永鹏、刘国秀、郦春锦、刘晓昱、赵思进、江砚、童芬美、阮菲、孙建宇、吴敏、陈红淑、吴瑾瑾、刘丽琴、陈占功、崔一然、范峥、李璐瑒、李洋、时琳、王莉、赵小伟、庄志宏、朱江、白小英、闫国强、韩永鹏、王春生、李培红、孔祥文、韩永龙、刘莉、郑敏霞、陈树和、赵学龙、王红丽、张立超、马传江、林华、年华、杨毅恒、彭康、张国豪、成彦武、章龙、刘史佳、汪洋、邵英俊、姜黎滨、杨玉州、李远龙、王庸全、张忠全、陈颖莉（中国香港）、张囡、李丝雨、林丽、钟长鸣、许岩、李亚楠、李易轩、孙茜茜、杨宛君、俞辰倩、曾凤萍、杨浣菲、杨凡林、李斐玉、郭梦蕊、尚静。

法　　国：Vassail Joëlle

美　　国：周传利

日　　本：赵鑫

审阅专家（排名不分先后）：

中　　国：姜黎滨、郭志武、陆芳、乔延江、徐雯洁、张海燕、刘红旭、王冠名（中国香港）

英　　国：江丹、汤淑兰

意 大 利：Rinaldo Rinaldi、Sergio Bangrazi、Vita Revelli

韩　　国：Seung Min Kathy Lee

加 拿 大：吴滨江

巴　　西：Reginaldo de Carvalho Silva Filho

斐　　济：涂志辉

日　　本：韩涛

本文件的起草程序遵守了世界中医药学会联合会发布的 SCM 1.1-2021《标准化工作导则 第 1 部分：标准制修订与发布》。

本文件由世界中医药学会联合会发布，版权归世界中医药学会联合会所有。

引　言

中药汤剂是中医治疗疾病的主要手段之一，在几千年防治疾病的用药实践中始终作为中药最重要的剂型发展运用，科学正确地服用汤剂直接影响中药临床疗效。世界卫生组织发布的《规范处方药物集》，对325种基本药物提供使用剂量、不良反应、禁忌症和注意事项；中国国家药品监督管理局颁布和修订《药品说明书和标签管理规定》，药品说明书得到充分发展和完善。

目前缺乏中药汤剂服用的指导规范，多数国家对于中药汤剂服用的指导工作尚未明确。例如，在法国，汤剂的服用交代一般由中医配药机构提供，以中医从业人员开具的方剂为准；在美国，汤剂的服用交代主要由给患者看病的针灸医师进行。因此，为提升中医药在全球范围内用药的安全性和有效性，迫切需要制定中药汤剂服用的指南，在中药汤剂发药指导时，为告知患者的服用提供参考依据。

本文件在系统梳理中药汤剂服用知识基础上，参考各国各地区相关法律法规，制定中药汤剂服用的知识体系框架和具体指导方法，以期促进世界范围内中药汤剂的规范服用。本文件若有任何与标准应用所在国的法律法规相抵触的内容，以该国的法律法规为依据。

常用中药汤剂服用指南

1 范围

本文件规定了常用中药汤剂服用的基本原则、通用性指导和个性化指导等内容。

本文件适用于中药汤剂服用人员，包括患者及患者家属；以及专业指导人员，包括医生、药师以及调剂员等。

2 规范性引用文件

下列文件中的内容通过文中的规范性引用而构成本文件必不可少的条款。其中，注日期的引用文件，仅该日期对应的版本适用于本文件；不注日期的引用文件，其最新版本（包括所有的修改单）适用于本文件。

SCM 55-2020 中药处方、调剂、给付与煎服要求 第 4 部分：中药煎服要求

T/CACM 1366-2021 中药汤剂煎煮规范

3 术语和定义

下列术语和定义适用于本文件。

3.1

中药饮片 Chinese medicine decoction pieces

中药饮片是指中药材经过加工炮制后可以直接用于中医临床或制剂生产使用的药品。

3.2

中药汤剂 Chinese medicine decoctions

中药饮片加水煎煮，去渣取汁内服的液体剂型；或中药配方颗粒等冲服后的液体制剂。

3.3

中药汤剂服法 Taking method of Chinese medicine decoctions

按时、按量、按疗程，科学合理服用中药汤剂的方法。

4 中药汤剂服用基本原则

4.1 患者宜根据年龄、性别、病情严重程度，遵循“随证施量、以知为度、获效停药”的原则服用中药汤剂。

4.2 中药汤剂服用指导应包括通用性指导和个性化指导两部分，患者及患者家属可参照

通用性指导内容执行，包括服用时间、服用次数、服用温度、服用剂量、服用疗程、贮存指导、特殊服法和自我记录 8 部分，框架参见附录 A.1；医师、药师、调剂员等专业从业人员在通用性指导基础上，酌加个性化指导内容，包括警示语、服用禁忌和药后护理等 3 部分，框架参见附录 A.2。指导内容宜遵循以下原则：

a）中药汤剂服用指导宜参照附录 B 的参考准则，从各国法律法规、经典教材和实践经验中提取知识。

b）常用中药汤剂的分类指导内容宜参照附录 C 给出的分类方法。

5 中药汤剂服用通用性指导

5.1 服用时间

5.1.1 中药汤剂的服用时间应符合 SCM 0055-2020 中“7.1.2”和“7.1.3”的规定。

5.1.2 治疗中医上焦疾病（指胸膈以上部位，包括心脏、肺部等）的中药汤剂，宜进餐后 30 分钟服用。

5.1.3 治疗中医下焦疾病（指腰腹以下部位，包括肝脏、肾脏、肠部等）的中药汤剂，宜进餐前 30~60 分钟服用。

5.1.4 治疗中医里证（指疾病表现于骨髓）的中药汤剂，宜晚餐后 30 分钟服用。

5.1.5 治疗中医表证（指疾病表现于四肢或血液）的中药汤剂，宜 8~10 小时内没有进食、次日晨起进食前服用。

5.1.6 具有消食功效或胃肠刺激性的中药汤剂，宜进餐后 30 分钟服用。

5.1.7 具有行、散、通利功效的中药汤剂，宜进餐前 30~60 分钟服用。

5.1.8 具有滋补、峻下逐水、攻积导滞、驱虫功效的中药汤剂，宜 8~10 小时内没有进食、次日晨起进食前服用。

5.1.9 治疗急病、重病的中药汤剂，可不按照特定的服用时间随时服药，或遵医嘱。

5.1.10 治疗慢性疾病的中药汤剂，宜定时服用，或遵医嘱。

5.2 服用次数

5.2.1 中药汤剂的服用次数应符合 SCM 0055-2020 中“7.1 通用要求”的规定。

5.2.2 治疗中医疾病表现为肺部疾病、哮喘、胃炎等夜间加重为主，宜间隔一定时间、昼夜连续服用多次。

5.2.3 治疗中医疾病表现出风寒湿邪致病或发作频繁，宜间隔一定时间、昼夜连续服用多次。

5.2.4 治疗中医疾病表现为急病、重病或以上焦病证为主，如呕吐、咽喉病等，宜少量多次服用。

5.2.5 治疗重症、中毒、猝死等疾病，宜 1 次快速服下。

5.2.6 治疗急症、高热类症状，宜 1 次快速服下。

5.2.7 药性峻猛、毒性剧烈或具有堕胎功效的中药汤剂，宜服用 1 次。

5.2.8 具有驱虫功效的中药汤剂，宜 1 次快速服下。

5.3 服用温度

5.3.1 中药汤剂的最佳服用温度以 30℃~37℃为宜，最高不可超过 65℃，或遵医嘱。

5.3.2 治疗中医疾病主要以高热病、出血热、中暑、便秘、热淋、呕吐、中毒等为主要表现，宜冷服、低于 26℃。
5.3.3 治疗中医疾病表现为急症、胃脘痛、内寒证、脾胃虚寒证，宜温服、37℃~40℃。
5.3.4 治疗中医疾病以真寒假热证为主，宜冷服、低于 26℃；
5.3.5 治疗中医疾病以真热假寒证为主，宜温服、37℃~40℃。
5.3.6 具有滋补、攻下作用的中药汤剂，宜温服、37℃~40℃。
5.3.7 具有止血、收涩、祛暑、解毒、止吐、清热功效的中药汤剂，宜冷服、低于 26℃。
5.3.8 具有辛温解表、理气、活血化瘀、祛寒、温通血脉类功效的中药汤剂，宜温服、37℃~40℃。

5.4 服用剂量

5.4.1 每日服用剂量以 1 日 1 剂为宜，头煎、二煎兑合，煎出液 400~600mL，分 2~3 次；200~300mL，分 1 次，或遵医嘱。
5.4.2 成人的每次服用剂量为 200~300mL；婴儿小于 1 岁服用，每次服用剂量以 1~5mL 为宜（药液宜浓缩）；1-2 岁幼儿服用，每次服用剂量以 20~40mL 为宜（药液宜浓缩）；3-7 岁学龄前儿童服用，每次服用剂量以 40~80mL 为宜；8~16 岁儿童服用，每次服用剂量以 100~150mL 为宜；如遇特殊情况，或遵医嘱。

5.5 服用疗程

5.5.1 治疗轻症疗程相对较短，治疗重症疗程相对较长，具体或遵医嘱。
5.5.2 治疗中医疾病为急病、重病，或以尿血、疼痛、泻下等为主要表现，或具有安神、活血、发汗等功效，见效停药，遵医嘱进行服药。
5.5.3 中药汤剂处方中含有砒石（红砒、白砒）、砒霜、水银、生马前子、生川乌、生草乌、生白附子、生附子、生半夏、生南星、生巴豆、斑蝥、青娘虫、红娘虫、生甘遂、生狼毒、生藤黄、生千金子、生天仙子、闹阳花、雪上一枝蒿、红升丹、白降丹、蟾酥、洋金花、红粉、轻粉、雄黄毒性中药品种，服药疗程尽量短，或遵医嘱。

5.6 贮存指导

5.6.1 针对各类中药房和中医诊所暂贮汤剂，应根据当地或医疗机构规定；宜于容器内煮沸、后进行袋装包装，以便贮存时间相对较长。
5.6.2 真空密封包装的汤剂宜放入冰箱冷藏室 2℃~8℃范围内保存，保存时间在两周内。
5.6.3 中药汤剂在室温通风下进行贮存，贮存时间不宜超过 3 天，且服用前应重新煮沸。
5.6.4 中药汤剂中含有全蝎、地龙、蜈蚣等动物类中药饮片，或含有熟地黄、黄芪、葛根等高糖高淀粉类中药饮片，宜放入冰箱冷藏室低温保存，保存时间不宜超过 7 天，且服用前应重新煮沸。
5.6.5 对于医疗机构代煎汤剂，若出现药液袋鼓起，或药液变色、变味、气泡等异常现象，不宜服用；对于自行煎煮汤剂，若贮存过程中出现变色、变味等异常现象，不宜服用。

5.7 特殊服法

5.7.1 “烊化”服用的中药应符合 T/CACM 1366-2021 中“9.2.2.4 烊化（溶化）”的规定。

5.7.2 “另煎”服用的中药应符合 T/CACM 1366-2021 中“9.2.2.3 另煎（另炖）”的规定。
5.7.3 “冲服”服用的中药应将药末合于已煎好的煎剂中搅拌服用。

5.8 自我记录

5.8.1 服用具有泻下或驱虫功效的中药汤剂，记录排便时间、次数及排便时的自我感觉。
5.8.2 服用具有利湿、逐水功效的中药汤剂，记录小便颜色、气味、次数以及尿量多少。
5.8.3 服用药性峻猛或含毒性饮片的中药汤剂，观察呕吐、腹痛等情况，若出现剧烈腹痛、呕吐不止、大汗淋漓、心慌气短等中毒现象，应立即停药并及时就医。
5.8.4 连续服用中药汤剂进行治疗时，定期监测肝肾功能，约每 3 月检查一次。

6 中药汤剂服用个性化指导

6.1 警示语

6.1.1 中药汤剂处方中含有砒石（红砒、白砒）、砒霜、水银、生马前子、生川乌、生草乌、生白附子、生附子、生半夏、生南星、生巴豆、斑蝥、青娘虫、红娘虫、生甘遂、生狼毒、生藤黄、生千金子、生天仙子、闹阳花、雪上一枝蒿、红升丹、白降丹、蟾酥、洋金花、红粉、轻粉、雄黄毒性中药品种，需注明警示内容。
6.1.2 其他见附录 B“警示语”。

6.2 服药禁忌

6.2.1 药物-药物

6.2.1.1 中药汤剂的相关配伍禁忌应符合 SCM 0055-2020 附录 C 中“C.1 配伍禁忌药”和“C.2 妊娠禁忌药”的规定。
6.2.1.2 中药汤剂与其他西药联合使用时，两种药物需间隔 0.5 小时或 1 小时以上。
6.2.1.3 其他见附录 B“药物-药物禁忌”。

6.2.2 药物-人群

6.2.2.1 中药汤剂中含有毒性饮片如砒石（红砒、白砒）、砒霜、水银、生马前子、生川乌、生草乌、生白附子、生附子、生半夏、生南星、生巴豆、斑蝥、青娘虫、红娘虫、生甘遂、生狼毒、生藤黄、生千金子、生天仙子、闹阳花、雪上一枝蒿、红升丹、白降丹、蟾酥、洋金花、红粉、轻粉、雄黄，儿童、肝肾功能不全患者慎用，妊娠期妇女禁用。
6.2.2.2 具有通经祛瘀、行气破滞功效的中药汤剂，或辛热燥烈、滑利通窍的中药汤剂，妊娠期妇女慎用。
6.2.2.3 具有活血化瘀、破血通络、通窍、泻下、逐水、止血功效的中药汤剂，月经期妇女慎用。
6.2.2.4 老年人在治疗过程与其他药物联用时不宜超过 3~4 种。
6.2.2.5 针对老年人群，60~69 岁中药用量应为成人量的 3/4，70~79 岁中药用量应为成人量的 3/5，80~89 岁中药用量应为成人量的 1/2，90 岁以上应仿效儿童剂量给药。

6.2.2.6 老年人禁止滥用滋补类中药汤剂。
6.2.2.7 肝肾功能不全患者避免具有同类功效的药物叠加使用。
6.2.2.8 其他见附录 B“药物-人群禁忌”。

6.2.3 药物-疾病

6.2.3.1 寒性疾病患者忌生冷；热性疾病患者忌辛热油腻。
6.2.3.2 脾胃虚弱、消化不良者忌食油炸、寒冷、坚硬不易消化食物。
6.2.3.4 肝阳上亢患者忌食胡椒、辣椒、大蒜，忌饮酒。
6.2.3.5 过敏体质患者忌食芒果、荔枝、花生及海产等发物。
6.2.3.6 皮肤病患者忌海产、羊肉等发物。
6.2.3.7 水肿病患者忌食腌制食品及盐。

6.2.4 药物-食物/保健品

6.2.4.1 中药汤剂的一般药物-食物禁忌包括忌生冷、辛辣、油腻及刺激性食物。
6.2.4.2 服用中药汤剂过程中忌茶、酒。
6.2.4.3 胸痹患者忌食肥肉、动物脏器。
6.2.4.4 具有发汗解表功效的中药汤剂忌酸味食物及冷饮。
6.2.4.5 具有清热凉血、解毒消肿、平肝、润肺、明目功效的中药汤剂，忌酒、蒜、咖啡、辣椒、羊肉等辛温刺激之品。
6.2.4.6 具有温经、补阳、涩精止泻、祛风湿、止寒痛功效的中药汤剂，忌冷饮、生梨、螃蟹、柿子、竹笋等寒凉之品。
6.2.4.7 具有养胃功效的中药汤剂忌糯米制食品。
6.2.4.8 具有逐水、利尿、利湿等功效的汤剂不宜加糖，具有温热性质的中药汤剂不宜加白糖，具有寒凉性质的中药汤剂不宜加红糖。
6.2.4.9 半夏茯苓汤治疗妊娠恶阻忌生冷醋滑油腻，菘菜，海藻；独活汤用于治疗恶风毒气，忌瓠子、蕺菜。
6.2.4.10 甘草汤治疗偏风积年不瘥服药过程中慎风冷、醋、蒜、面、乳、酪、鱼等。

6.2.5 药物-环境

6.2.5.1 具有安胎功效的中药汤剂服用后宜温卧，保持室内环境安静。
6.2.5.2 具有祛风湿功效的中药汤剂服用后不宜居住在潮湿环境，或避免水下作业。
6.2.5.3 治疗风邪致病的中药汤剂服用后需要避风。
6.2.5.4 其他见附录 B“药物-环境禁忌”。

6.3 服药后护理

6.3.1 护理方式

6.3.1 对于医嘱需“啜热稀粥”进行辅助药效发挥的汤剂，宜在服药后服少量热粥（60℃以上为宜）。
6.3.2 对于医嘱需“温覆”辅助药效发挥的汤剂，服药后宜盖好被子静卧。

6.3.3 对于医嘱需“见效停药”等的汤剂，若出现指标及症状改善情况应立即停药。
6.3.4 具有催吐功效的中药汤剂吐而不止时，宜服用姜汁、冷稀粥（30℃为宜）或凉开水止吐。
6.3.5 具有安胎功效的中药汤剂，服药后宜适当温卧。
6.3.6 中药汤剂治疗胃肠道疾病时，服药后宜适当温卧。

6.3.2 可能产生的不良反应

6.3.2.1 误服或超剂量服用中药汤剂，可能出现头晕、恶心、中毒性休克等毒性反应，应立即停药并进行抢救。
6.3.2.2 服用含生物碱类成分中药饮片的中药汤剂，服药后可能出现中枢神经系统、自主神经系统相关中毒表现，如语言不清、恶心呕吐、呼吸困难、心率失常等。
6.3.2.3 服用含毒性蛋白类成分中药饮片的中药汤剂，服药后可能出现消化系统及神经功能障碍相关中毒表现，如红细胞溶解、全身广泛性出血等。
6.3.2.4 服用含毒苷中药饮片的中药汤剂，服药后可能中枢神经、心脏相关中毒表现，如便血、昏厥、四肢麻木、呼吸停止、心源性脑供血不足等。
6.3.2.5 服用含萜类与内酯类中药饮片的中药汤剂，服药后可能出现消化道和肝脏相关中毒表现，如全身麻木、胸闷、剧烈呕吐等。
6.3.2.6 服用含动物中药饮片的中药汤剂，服药后可能出现肝脏、肾脏和心血管相关中毒表现，如便血、双下肢瘫痪、大小便困难、呕血等。
6.3.2.7 服用含矿物类类中药饮片的中药汤剂，服药后可能出现神经系统、造血系统、消化系统、心血管系统以及肝脏、肾脏等相关中毒表现，如呼吸困难、头晕目眩、机头痉挛等。
6.3.2.8 其他见附录 B“可能产生的不良反应”。

6.3.3 服药后对排泄物产生的影响

服用含栀子、丹参、牛黄、大黄、番泻叶、芦荟、黄连、黄芩饮片的中药汤剂可能使尿液发生符合表 1 给出的颜色变化，停药后症状可自行消失；如有不适，应立即停止服药并就医。

表 1 服药后可能对排泄物产生的影响

含中药饮片的汤剂	对尿液颜色产生的影响
栀子、丹参	红色
牛黄	黄色
大黄、番泻叶、芦荟	深黄色
黄连、黄芩	黄绿色

6.3.4 用药监测

6.3.4.1 具有泻下或驱虫功效的中药汤剂，需监测大便次数、质量、颜色、气味以及是否有虫体排除，若大便次数和数量较多，可停止服药；若服药后 3~4 小时仍无便意，可增加服药剂量。
6.3.4.2 具有利湿、逐水功效的中药汤剂，需监测小便颜色、气味、次数以及尿量的多少，

若小便次数和数量较多，可停止服药。

6.3.4.3 中药汤剂药性峻猛或含毒性饮片，需监测血压、呕吐、腹痛等情况。

附录 A
（规范性）
中药汤剂服用指南的知识框架

发药时，药师对患者进行的中药汤剂服用指导应包含通用性指导和个性化指导等两部分内容，根据患者的性别、年龄、体质、所患疾病的病位、病势、病机以及严重程度的不同有针对性地进行服药指导，主次分明。其中，患者参看的服用指导可见图 A.1，医师/药师等专业从业人员参看的服用指导可见图 A.2。

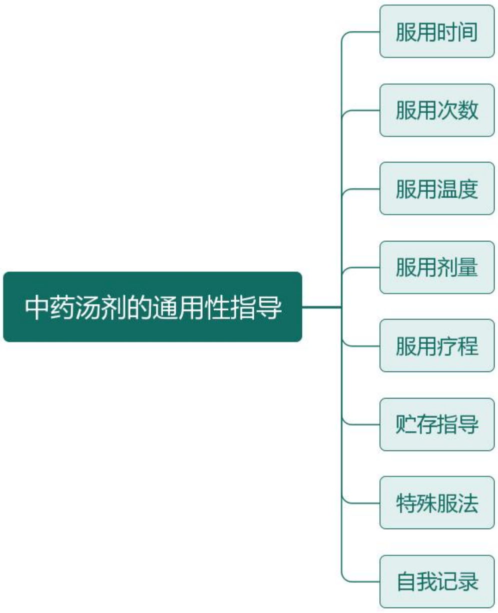

图 A.1 中药汤剂的通用性指导知识框架

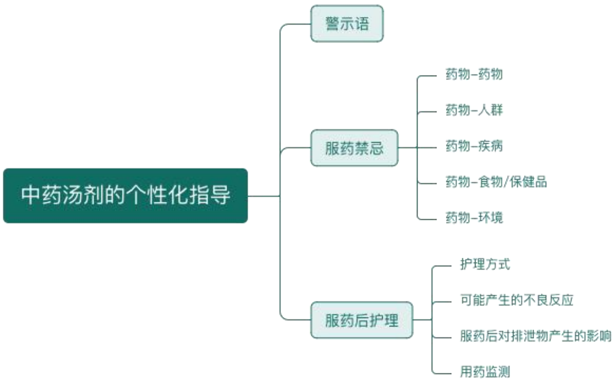

图 A.2 中药汤剂的个性化指导知识框架

附录 B

（资料性）

中药汤剂服用指南知识框架的参考准则

中药汤剂服用知识体系框架包括“中药汤剂的名称和基本治疗功效”等 7 个一级知识条目，“中药汤剂的服用时间”等 13 个二级知识条目和“《中国药典》（2020 年版）”等 82 个参考准则。在中药汤剂服用指南知识体系下，可参考现有的中医药法规、标准、教材等文件提取 82 条知识内容（见表 B.1）。

表 B.1 中药汤剂服用指南二级要素及其参考准则

一级要素	二级要素	参考准则	知识内容编号
1 名称及基本功效	——	1-古代中医典籍《伤寒杂病论》《备急千金要方》	1
		2-《方剂学》（李冀，左铮云主编，中国中医药出版社，2021）	2
2 服用方法（包含中药饮片特殊服法的告知，如烊化等）	1.服用时间	1-中华医典（第 5 版）	3
		2-《中医内科常见病诊疗指南》	4
		3-《方剂学》（李冀，左铮云主编，中国中医药出版社，2021）	5
	2.服用次数	1-中华医典（第 5 版）	6
		2-《中医内科常见病诊疗指南》	7
		3-《方剂学》（李冀，左铮云主编，中国中医药出版社，2021）	8
		4-临床随机对照试验	9
	3.服用温度	1-中华医典（第 5 版）	10
		2-《中医内科常见病诊疗指南》	11
		3-《方剂学》（李冀，左铮云主编，中国中医药出版社，2021）	12
	4.服用剂量	1-中华医典（第 5 版）	13
		2-《中医内科常见病诊疗指南》	14
		3-《方剂学》（李冀，左铮云主编，中国中医药出版社，2021）	15
		4-临床随机对照试验	16
	5.服用疗程（有即告知）	1-中华医典（第 5 版）	17
		2-《中医内科常见病诊疗指南》	18
		3-《方剂学》（李冀，左铮云主编，中国中医药出版社，2021）	19
		4-临床随机对照试验	20
中药汤剂的贮存条件	——	1-临床实际用药经验（根据地域和医疗机构不同）	21

警示语	——	1-《中国药典》2020 年版	22
		2-《中华人民共和国药典临床用药须知中药卷》（国家药典委员会，人民卫生出版社，2020）	23
		3-《国家基本药物临床应用指南》（国家基本药物临床应用指南编委会，人民卫生出版社，2010）	24
		4-国内外期刊数据库	25
中药汤剂的服用禁忌	1.药物-药物（包含中-西药联用）	1-药品不良反应监测数据库	26
		2-《中国药典》2020 年版	27
		3-《中华人民共和国药典临床用药须知中药卷》（国家药典委员会，人民卫生出版社，2020）	28
		4-药品上市许可持有人收集的临床前研究、上市前临床试验及上市后再评价研究	29
		5-《临床中药药物治疗学》（张冰,周祯祥主编，人民卫生出版社，2016）	30
		6-国内外期刊数据库	31
		7-中华医典（第 5 版）	32
		8-《中成药与西药的相互作用》（曹俊岭，甄汉深主编，人民卫生出版社，2016）	33
	2.药物-人群	1-药品不良反应监测数据库	34
		2-《中华人民共和国药典临床用药须知中药卷》（国家药典委员会，人民卫生出版社，2020）	35
		3-《临床中药药物治疗学》（张冰,周祯祥主编，人民卫生出版社，2016）	36
		4-国内外期刊数据库	37
		5-中华医典（第 5 版）	38
		6-临床实际用药经验	39
	3.药物-食物/保健品	1-药品不良反应监测数据库	40
		2-《中国药典》2020 年版	41
		3-《中华人民共和国药典临床用药须知中药卷》（国家药典委员会，人民卫生出版社，2020）	42
		4-药品上市许可持有人收集的临床前研究、上市前临床试验及上市后再评价研究	43
		5-《临床中药药物治疗学》（张冰,周祯祥主编，人民卫生出版社，2016）	44
		6-国内外期刊数据库	45
		7-中华医典（第 5 版）	46
	4.药物-疾病	1-药品不良反应监测数据库	47
		2-《中国药典》2020 年版	48

		3-《中华人民共和国药典临床用药须知中药卷》（国家药典委员会，人民卫生出版社，2020）	49
		4-药品上市许可持有人收集的临床前研究、上市前临床试验及上市后再评价研究	50
		5-《临床中药药物治疗学》（张冰,周祯祥主编，人民卫生出版社，2016）	51
		6-国内外期刊数据库	52
		7-中华医典（第 5 版）	53
	5.药物-环境	1-药品不良反应监测数据库	54
		2-《中国药典》2020 年版	55
		3-《中华人民共和国药典临床用药须知中药卷》（国家药典委员会，人民卫生出版社，2020）	56
		4-药品上市许可持有人收集的临床前研究、上市前临床试验及上市后再评价研究	57
		5-《临床中药药物治疗学》（张冰，周祯祥主编，人民卫生出版社，2016）	58
		6-国内外期刊数据库	59
		7-《中国药典》2020 年版	60
中药汤剂的药后护理	1.服药后恰当的药后护理方式	1-中华医典（第 5 版）	61
		2-《中医内科常见病诊疗指南》	62
		3-《中药药物警戒》（张冰主编，人民卫生出版社，2015）	63
		4-《中医内科学》（吴勉华，石岩主编，中国中医药出版社，2021）	64
		5-《中华人民共和国药典临床用药须知中药卷》（国家药典委员会，人民卫生出版社，2020）	65
		6-国内外期刊数据库	66
		7-药品上市许可持有人收集的临床前研究、上市前临床试验及上市后再评价研究	67
		8-《临床中药药物治疗学》（张冰，周祯祥主编，人民卫生出版社，2016）	68
		9-临床实际用药经验	69
	2.不良反应的预防/救治措施（包含可能产生的不良反应）	1-中华医典（第 5 版）	70
		2-《中医内科常见病诊疗指南》	71
		3-《中药药物警戒》（张冰主编，人民卫生出版社，2015）	72
		4-《中华人民共和国药典临床用药须知中药卷》（国家药典委员会，人民卫生出版社，2020）	73
		5-药品上市许可持有人收集的临床前研究、	74

		上市前临床试验及上市后再评价研究	
		6-国内外期刊数据库	75
		7-《临床中药药物治疗学》（张冰，周祯祥主编，人民卫生出版社，2016）	76
		8-临床实际用药经验	77
	3.用药记录/自我监测	1-临床实际用药经验	78
		2-国内外期刊数据库	79
	4.服药对排泄物产生的影响	1-中华医典（第 5 版）	80
		2-临床实际用药经验	81
		3-《中药药物警戒》（张冰主编，人民卫生出版社，2015）	82

附录 C

（资料性）

常用中药汤剂示例

C.1 常用中药汤剂示例索引

本部分内容按照疾病分类法对国家中医药管理局 2018 年发布的《古代经典名方目录（第一批）》，东汉·张仲景所著的《伤寒杂病论》，唐·孙思邈所著的《备急千金要方》中的中药汤剂处方进行索引研究，筛选方剂名称、处方组成、主治病症、服药温度、服药时间、服药次数、服药剂量、疗程、服药禁忌、药后护理、疗效观察对应部分内容记录清晰者；且处方中不包含《中华人民共和国药典》（2020 年版）收载的大毒中药，不涉及国家重点保护的野生动物药材品种目录的一级保护品种；处方为常用中药或中药饮片处方，其中不含有十八反、十九畏等配伍禁忌。共筛选常用中药汤剂示例 461 首。

病证分类	类型		常用方剂
肺系疾病	咳嗽	诸气咳嗽	厚朴汤
		肺气上咳	橘皮汤
		肺气不足	补肺汤
		肺气不足，腰痛	小建中汤
		咳嗽	桂苓五味甘草汤
			苓甘五味姜辛汤
			桂苓五味甘草去桂加姜辛半夏汤
			加姜辛半夏杏仁汤方
			苓甘五味加姜辛半杏大黄汤
			小青龙加石膏汤
			射干麻黄汤
			厚朴麻黄汤
			麦门冬汤
			麻黄石膏汤
			十枣汤
			温脾汤
			百部根汤
			海藻汤
			白前汤
			竹皮汤
	肺痈	——	桔梗汤
			泻肺汤
	肺劳	肺气虚重症	麻黄引气汤
			半夏汤
			厚朴汤
			黄芪汤
			大前胡汤

			竹叶汤
			桂枝去芍药加皂荚汤
	小儿癖结胀满	——	紫菀汤
			五味子汤
			射干汤
			桂枝汤
	肺痿	——	甘草干姜汤
			甘草汤
	小儿伤寒	——	麦门冬汤
			芍药四物解肌汤
			麻黄汤
			五味子汤
			升麻汤
			大黄汤
			竹叶汤
			调中汤
			生地黄汤
			恒山汤
	伤寒		桂枝汤
			桂枝加桂汤
			桂枝加芍药汤
			桂枝加大黄汤
			桂枝加厚朴杏子汤
			桂枝加附子汤
			桂枝去芍药汤
			桂枝去芍药加附子汤
			桂枝甘草龙骨牡蛎汤
			桂枝人参汤
			桂枝附子汤
			桂枝去桂加茯苓白术汤
			桂枝加芍药生姜各一两人参三两新加汤
			桂枝麻黄各半汤
			麻黄汤
			大青龙汤
			小青龙汤
			桂枝二麻黄一汤
			桂枝二越婢一汤
			麻黄细辛附子汤
			麻黄附子甘草汤
			麻黄杏仁甘草石膏汤
			葛根汤
			葛根加半夏汤
			葛根黄芩黄连汤

			桂枝加葛根汤
			白虎汤
			白虎加人参汤
			竹叶石膏汤
			调胃承气汤
			小承气汤
			大承气汤
			桃核承气汤
			大陷胸汤
			小陷胸汤
			栀子豉汤
			栀子甘草豉汤
			栀子生姜鼓汤
			栀子厚朴汤
			栀子干姜汤
			栀子柏皮汤
			茵陈蒿汤
			枳实栀子豉汤
			小柴胡汤
			大柴胡汤
			柴胡加芒硝汤
			柴胡桂枝汤
			柴胡桂枝干姜汤
			黄芩汤
			黄芩加半夏生姜汤
			大黄黄连泻心汤
			附子泻心汤
			甘草泻心汤
			半夏泻心汤
			生姜泻心汤
			干姜黄芩黄连人参汤
			黄连汤
			旋复代赭汤
			四逆汤
			通脉四逆汤
			通脉四逆加猪胆汁汤
			干姜附子汤
			四逆加人参汤
			茯苓四逆汤
			附子汤
			真武汤
			白通汤
			当归四逆汤

			当归四逆加吴茱萸生姜汤
			甘草汤
			桔梗汤
			桂枝甘草汤
			甘草干姜汤
			芍药甘草汤
			芍药甘草附子汤
			茯苓甘草汤
			茯苓桂枝甘草大枣汤
			茯苓桂枝白术甘草汤
			厚朴生姜半夏甘草人参汤
			小建中汤
			炙甘草汤
			赤石脂禹余粮汤
			桃花汤
			猪苓汤
			吴茱萸汤
			麻黄升麻汤
			甘草附子汤
			白头翁汤
			桂枝附子去桂加白术汤
			桂枝芍药知母汤
			黄芪桂枝五物汤
			半夏厚朴汤
			瓜蒌薤白半夏汤
			苓桂术甘汤
			泽泻汤
			百合地黄汤
			枳实薤白桂枝汤
			橘皮竹茹汤
			麦门冬汤
			甘姜苓术汤
			厚朴七物汤
			厚朴麻黄汤
			当归建中汤
			温脾汤
			温胆汤
			小续命汤
			三化汤
心系病证	心悸	子烦（妊娠中期烦躁心悸）	竹沥汤
		心气弱悸	大补心汤
		风虚惊悸	远志汤

			茯神汤
			补心汤
			小定心汤
			大定心汤
			荆沥汤
			镇心汤
	胸痹	心实热，烦闷	石膏汤
		心下痞满	泻心汤
		心痛	桂心三物汤
			五辛汤
			高良姜汤
			温中当归汤
			羊肉当归汤
			温脾汤
		胸痹	瓜蒌汤
			枳实薤白桂枝汤
			茯苓汤
			通气汤
			前胡汤
			槟榔汤
	心劳热	——	大黄泻热汤
			升麻汤
			麻黄调心泄热汤
	其他病证	心气不足，吐血	大黄黄连泻心汤
		惊梦喜笑恐畏悸惧不安	竹沥汤
		心气不足	茯苓补心汤
		心虚寒，心中胀满	半夏补心汤
脑系病证	眩晕	风眩	防风汤
	中风	产后中风	大豆紫汤
			独活紫汤
			小独活汤
			甘草汤
			独活汤
			竹叶汤
			防风汤
			独活汤
			大豆汤
			五石汤
			四石汤
			小柴胡汤
			甘草汤
			葛根汤
			茯神汤

			远志汤
			茯苓汤
			安心汤
		中风	小续命汤
			大续命汤
			西州续命汤
			排风汤
			大八风汤
			乌头汤
			大防风汤
		贼风中风	川芎汤
			依源麻黄续命汤
			温中生姜汤
			乌头汤
			防风汤
			羌活汤
			防己汤
			大枣汤
		偏风	防风汤
			葛根汤
			仲景三黄汤
			白蔹薏苡汤
			独活寄生汤
		中风后偏瘫	竹沥汤
		严重中风昏迷	独活汤
		角弓反张	仓功当归汤
		心系病证-头面风	茯神汤
			犀角汤
	痫证	小儿风痫	大黄汤
			增损续命汤
			石膏汤
脾胃系病证	呕吐	妊娠恶阻	半夏茯苓汤
			青竹茹汤
			橘皮汤
		胃不受食，食即呕吐	大半夏汤
		呕吐哕逆	半夏汤
			前胡汤
			小麦汤
			橘皮汤
			大黄甘草汤
	噎嗝	噎塞	竹皮汤
			干姜汤
			通气汤

	腹痛	产后腹痛	芍药汤
			当归汤
			羊肉汤
			羊肉当归汤
			羊肉杜仲汤
			羊肉地黄汤
			内补当归建中汤
			内补川芎汤
			大补中当归汤
			吴茱萸汤
			蒲黄汤
			败酱汤
			独活汤
			芍药黄芪汤
	泄泻	产后下痢	白头翁汤
			鳖甲汤
			干地黄汤
			生地黄汤
	痢疾	热痢	三黄白头翁汤
			白头翁汤
			茯苓汤
		冷痢	温脾汤
			大桃花汤
			仓米汤
			附子汤
			断痢汤
			泻心汤
			香苏汤
		小儿痢	温中汤
			温中大黄汤
			大黄汤
	便秘	——	三黄汤
	其他病证	脾热黄疸	大黄泻热汤
		脾劳	半夏汤
		口苦	补胃汤
		胃胀满	温胃汤
			大半夏汤
			附子粳米汤
			厚朴三物汤
			厚朴七物汤
			吴茱萸汤
			大桂汤
		痼冷积热	半夏汤

			生姜汤
			甘草汤
			茱萸硝石汤
			大黄附子汤
			大乌头汤
			五石汤
			竹叶汤
			半夏汤
			承气汤
肝胆系病证	胁痛	产后胁痛	干地黄汤
		肝气不足，胁满	补肝汤
		肝虚寒，胀满胁痛	槟榔汤
	积聚	坚症积聚	陷胸汤
			甘遂汤
	其他病证	小儿痈疽瘰疬	漏芦汤
			麻黄汤
		手脚抽筋	橘皮通气汤
肾系疾病	水肿	妊娠水肿，小便不利	鲤鱼汤
	其他病证	产后淋渴	瓜蒌汤
			竹叶汤
		肾实热小腹胀满	泻肾汤
		肾劳实热	栀子汤
		精气衰竭	竹叶黄芩汤
		骨极	三黄汤
		腰痛	肾着汤
气血津液病证	吐血	——	生地黄汤
			坚中汤
			柏叶汤
			泽兰汤
			犀角地黄汤
			当归汤
			竹茹汤
			麦门冬汤
	痰饮	——	小半夏汤
			甘草汤
			木防己汤
			厚朴大黄汤
			小半夏加茯苓汤
			甘遂半夏汤
			大茯苓汤
			茯苓汤
			大半夏汤
			半夏汤

			当归汤
			吴茱萸汤
			前胡汤
			旋覆花汤
			姜椒汤
			断膈汤
	虚劳	产后虚损	羊肉汤
			猪肾汤
			羊肉黄芪汤
			当归芍药汤
			杏仁汤
			五石汤
			三石汤
			内补黄芪汤
			吴茱萸汤
			鲤鱼汤
			桂枝加附子汤
		产后虚烦	薤白汤
			人参当归汤
			甘竹茹汤
			知母汤
			竹叶汤
			淡竹茹汤
			芍药汤
		胆气虚导致虚烦	温胆汤
			酸枣汤
			栀子汤
		虚劳	建中汤
			小建中汤
			黄芪建中汤
			前胡建中汤
			乐令建中汤
			黄芪汤
			大建中汤
			肾沥汤
			增损肾沥汤
			凝唾汤
			人参汤
肢体经络病证	痹证	风毒脚气	第一竹沥汤
			第三竹沥汤
			麻黄汤
			第二服独活汤
			第四服风引独活汤兼补方

			独活汤
			越婢汤
			风引汤
			犀角旋复花汤方
			大犀角汤
			吴茱萸汤
			小风引汤
			四物附子汤
			半夏汤
			紫苏子汤
			附子汤
			防风汤
			甘草汤
	痿证	——	解风痹汤
			西州续命汤
七窍病证	目病	视物不清	泻肝汤
	鼻病	流鼻血	生地黄汤
其他疾病	——	不孕不育	朴硝荡胞汤
		安胎	乌雌鸡汤方
			补胎汤
			艾叶汤方
			黄连汤
			雄鸡汤方
			茯神汤
			菊花汤方
			安中汤方
			麦门冬汤方
			柴胡汤
			葱白汤方
			杏仁汤
			芍药汤
			半夏汤
			猪肾方
		胎动不安	葱白汤
			旋复花汤
			胶艾汤
		下乳	单行鬼箭汤
		恶露	干地黄汤
			泽兰汤
			甘草汤
			大黄汤

C.2 代表示例：桂枝汤的服用指南

C.2.1 基础功效

桂枝汤功能解肌发表，调和营卫。主治外感风寒表虚证。恶风发热，汗出头痛，鼻鸣干呕，苔白不渴，脉浮缓或浮弱。

C.2.2 通用性指导

C.2.2.1 服用时间

宜空腹服。

C.2.2.2 服用次数

根据病情变化灵活掌握桂枝汤的服用次数，一日 1-3 次。

C.2.2.3 服用温度

37℃～40℃为宜。

C.2.2.4 中药汤剂的服用剂量

成人每次剂量 200mL 为宜，老人、儿童、孕妇及哺乳期妇女请遵医嘱服药。

C.2.2.5 中药汤剂的服用疗程

桂枝汤的服用疗程与病情变化相关，若一服药后汗出病情好转，可以停药；如果未出汗，继续服用；如果还未出汗，1 日内服尽 3 剂；若患者病情较重，服药时密切关注病情变化，可相对延长服药疗程；若一直未出现发汗情况，需连续服用 2-3 天，或遵医嘱。

C.2.2.6 中药汤剂的贮存条件

真空密封包装的汤剂宜放入冰箱冷藏室 2-8℃范围内保存，保存时间在两周内；若服用过程中出现药液袋鼓起，或药液变色、变味、气泡等异常现象，不宜服用。

C.2.3 个性化技术指导

C.2.3.1 中药汤剂的服用禁忌

C.2.3.1.1 中药汤剂的药物-药物禁忌（包含中西药联用）

桂枝汤中含有甘草，服用此方的同时不可服用海藻、大戟、芫花、甘遂饮片或含其饮片的中成药或成方制剂；与强心苷类药物地高辛、洋地黄同用时，注意有无地高辛中毒反应发生；与降压药利血平、降糖药、酸性西药、酶制剂、抗菌药红霉素、四环素、氯霉素、华法林、香豆素类抗凝药、水杨酸衍生物类药、排钾类利尿药、卡马西平、甲基多巴、H2 受体阻断剂、维生素 A 等药物同用，均可能降低其疗效；与三环素类抗抑郁药同用时，可能使三环素类抗抑郁药代谢产物增多，引发不良反应。

患者在服用桂枝汤的过程中不宜加糖。

若出现汤剂焦煎的情况，不宜服用。

C.2.3.1.2 中药汤剂的药物–人群禁忌

桂枝辛温助热，温通经脉，孕妇及月经过多者需在医生指导下进行服用。

生姜助火伤阴，热盛及心虚内热者不宜服用桂枝汤。

食积、虫积、龋齿作痛、痰热咳嗽的患者不宜服用桂枝汤。

长期饮酒或酗酒的患者不宜服用桂枝汤。

C.2.3.1.3 中药汤剂的药物–疾病禁忌

桂枝辛温助热，易伤阴动血，外感热病、阴虚火旺、血热妄行、湿盛脘腹胀满等证不宜服用桂枝汤。

外感风寒表实无汗者禁用桂枝汤。

C.2.3.1.4 中药汤剂的药物-食物/保健品禁忌

服用桂枝汤的过程中需禁生冷、黏滑、肉面、五辛、酒酪、臭恶等物。

C.2.3.1.5 中药汤剂的药物-环境禁忌

服用桂枝汤的过程中需注意避风。

C.2.3.2 中药汤剂的药后护理

服用桂枝汤后，啜热稀粥一升余。温覆令一时许，遍身热染微似有汗者益佳。

C.2.3.3 中药汤剂服用后对排泄物产生的影响

观察发汗状态，不可令如水流漓。

C.2.3.4 用药监测

监测病情变化和发汗程度。

参考文献

[1] 世界中医药学会联合会.SCM 0008-2011 国际中医医师专业技术职称分级标准, 人民卫生出版社, 2011.

[2] FIP GUIDELINES FOR THE LABELS OF PRESCRIBED MEDICINE，2001，Singapore

[3] SCM 0055-2020 中药处方、调剂、给付与煎服要求 第 4 部分：中药煎服要求

[4] T/CACM 1366-2021 中药汤剂煎煮规范

[5] T/CACM 1370.1—2021 上市中成药说明书安全信息项目修订技术规范 通则

[6] 翟华强, 黄晖, 郑虎占. 实用中药临床调剂技术[M]. 北京:人民卫生出版社. 2011. 8.

[7] 刘起华, 仝小林. 汤剂煎服法研究[M]. 北京:科学出版社. 2016. 6.

[8] 李国华. 为现代医师译经典《伤寒论》分册[M]. 北京:中医古籍出版社. 2013. 6.

[9] 王斌, 吴剑坤. 图解千金方[M]. 江苏:江苏凤凰科学技术出版社. 2020. 1.

[10] 中国国家药典委员会. 中华人民共和国药典（2020 版, 一部）[M]. 北京:中国医药科技出版社.

[11] 李冀, 左铮云. 方剂学（“十四五”规划教材）[M]. 北京:中国中医药出版社, 2021.

[12] 李庆业, 高琳, 王云阁, 赵晖, 杨桢. 汤头歌诀白话解（第 6 版）[M]. 北京:人民卫生出版社. 2016.

[13] 李培生. 伤寒论讲义（供中医专业用）[M]. 上海:上海科学技术出版社, 2017. 4.

[14] 国家基本药物临床应用指南编委会. 国家基本药物临床应用指南[M]. 北京:人民卫生出版社, 2011.

[15] 张冰, 周祯祥. 临床中药药物治疗学[M]. 北京:人民卫生出版社, 2016.

[16] 曹俊岭, 甄汉深. 中成药与西药的相互作用[M]. 北京:人民卫生出版社, 2016.

[17] 吴勉华, 石岩. 中医内科学 [M]. 北京:中国中医药出版社, 2021.

[18] 张冰. 中药药物警戒[M]. 北京:人民卫生出版社, 2015.

[19] 宜华. 说说中药的保质期[J]. 江苏卫生保健, 2021(06): 44.

[20] 郑弘, 许红玮, 秦静海, 等. 汤剂包装机不同的处理方法对中药汤剂保质期的影响[J]. 中医研究, 2014, 27(03): 70-72.

[21] 张立双, 江丰, 金鑫瑶, 等. 基于文献的中药汤剂服药方法分析[J].环球中医药, 2018, 11(12): 1946-1949.

[22] 翟华强, 王燕平. 临床常用方剂与中成药[M]. 北京:人民卫生出版社, 2020.

[23] 李玉峰, 张寿山, 郭蓉娟, 翟华强.内科疾病药物合理联用处方[M]. 郑州: 河南科学技术出版社, 2021.

Foreword

Some of the contents of this document may involve related patents, and the issuing organization of this document is not responsible for identifying the patents.

Main drafting unit: Beijing University of Traditional Chinese Medicine, Hangzhou Hospital of Traditional Chinese Medicine, Centre France China de la Médecine Chinoise (CFCMC), Beijing Hospital of Traditional Chinese Medicine, Capital Medical University Zhejiang Provincial People's Hospital.

Participants: Beijing Hospital of Integrative Medicine, Hangzhou Tanggu Information Technology Co. Confédération Française de Médécine Traditionnelle Chinoise (CFMTC), French KNP Group, Daiyu Group (USA), Naimoto Corporation (Japan), Hong Kong Chinese Medicine Hospital Development Program Office, Zhong Guan Cun Hospital of the Chinese Academy of Sciences, Beijing Society of Traditional Chinese Medicine, Xiyuan Hospital of the Chinese Academy of Traditional Chinese Medicine, The Third Affiliated Hospital of the University of Traditional Chinese Medicine, Beijing, Zhejiang Provincial Hospital of Traditional Chinese Medicine, Zhejiang Provincial Hospital of Integrative Medicine, Shanghai Sixth People's Hospital, Chengdu University of Traditional Chinese Medicine Hospital, Hubei Provincial Hospital of Traditional Chinese Medicine, Nanjing Hospital of Traditional Chinese Medicine, Gansu Provincial Hospital of Traditional Chinese Medicine, Shanghai Municipal Hospital of Traditional Chinese Medicine, Shandong Provincial Hospital of Traditional Chinese Medicine, Guangdong Provincial Hospital of Traditional Chinese Medicine, Shanghai University of Traditional Chinese Medicine's Yueyang Hospital of Integrative Medicine, Peking University's Third Hospital, and Nanfang Medical University's Hospital of Integrative Medicine, Shanghai Pudong New District Guangming Hospital of Traditional Chinese Medicine, Qingdao Hospital of Traditional Chinese Medicine, Cangzhou Hospital of Combined Traditional Chinese and Western Medicine, Beijing Institute of Health Professions, Zhejiang University of Traditional Chinese Medicine Chinese Medicine Drinking Tablets Co. Beijing Huamiao Pharmaceutical Co.

Experts of the Steering Committee: Wang Yongyan, Jin Shiyuan, Qiao Yanjiang, Wang Yanping, Zhang Huamin.

Main drafters: Zhai Huaqiang, Chen Hongmei, Wu Wanlin (France), Wu Jiankun, Zhang Guobing.

Participating drafters:

China: Han Yongpeng, Liu Guoxiu, Li Chunjin, Liu Xiaoyu, Zhao Sijin, Jiang Yan, Tong Fenmei, Ruan Fei, Sun Jianyu, Wu Min, Chen Hongshu, Wu Jinjin, Liu Liqin, Chen Zhanguong, Cui Yiran, Fan Zheng, Li Lujian, Li Yang, Shi Lin, Wang Li, Zhao Xiaowei, Zhuang Zhihong, Zhu Jiang, Bai Xiaoying, Yan Guoqiang, Wang Chunsheng, Li Peihong, Kong Xiangwen, Han Yonglong, Liu Li, Zhen Minxia, Chen Shuho, Zhao Xuelong, Wang Hongli, Zhang Lichao, Ma Chuanjiang, Lin Hua, Nian Hua, Yang Yiheng, Peng Kang, Zhang Guohao, Cheng Yanwu, Zhang Long, Liu Shijia, Wang Yang, Shao Yingjun, Jiang Libin, Yang Yuzhou, Li Yuanlong, Wang Yongquan, Zhang Zhongquan, Chen Yingli (Hong Kong, China), Zhang Nannan, Li Shiyu, Lin Li, Zhong Changming, Xu Yan, Li Yanan, Li Yixuan, Sun Xixi, Yang Wanjun, Yu Chenqian, Zeng Fengping, Yang Ruanfei, Yang Fanlin, Li Feiyu, Guo Mengrui, Shang Jing.

France: Vassail Joëlle

USA: Chuanli Zhou

Japan: Xin Zhao

Reviewing Experts (in no particular order):

China: Jiang Libin, Guo Zhiwu, Lu Fang, Qiao Yanjiang, Xu Wenjie, Zhang Haiyan, Liu Hongxu, Wang Guanming (Hong Kong, China)

UK: Jiang Dan, Yang Shulan

Italy: Rinaldo Rinaldi、Sergio Bangrazi、Vita Revelli

Korea: Seung Min Kathy Lee

Canada: Wu Binjiang

Brazil: Reginaldo de Carvalho Silva Filho

Fiji: Tu Zhihui

Japan: Han Tao

The drafting process of this document adheres to SCM 1.1-2021, "Guidelines for Standardization Part 1: Standard Formulation, Revision and Publication" issued by the World Federation of Chinese Medicine Societies (WFTC).

This document is issued by the World Federation of Chinese Medicine Societies and is copyrighted by the World Federation of Chinese Medicine Societies.

Introduction

Chinese medicine decoctions is one of the main means of Chinese medicine treatment of disease, in thousands of years of disease prevention and treatment of medication practice has always been as the most important preparation form of traditional Chinese medicine development and utilization, scientific and correct consumption of Chinese medicine decoctions directly affect the clinical efficacy of traditional Chinese medicine. The World Health Organization issued the "standardized prescription drug set", 325 kinds of basic drugs to provide the use of dosage, adverse reactions, contraindications and precautions; China National Medical Products Administration issued and revised the "Drug Description and Labeling Regulations", the drug specification has been fully developed and improved.

There is a lack of guidance specifications for the administration of Chinese medicine decoctions, and guidance for the administration of Chinese medicine decoctions has not been clarified in most countries. For example, in France, the accounting for the taking of decoctions is generally provided by Chinese medicine dispensing organizations, based on prescriptions issued by Chinese medicine practitioners; in the United States, the accounting for the taking of decoction is mainly carried out by acupuncturists who see patients. Therefore, in order to enhance the safety and efficacy of the use of Chinese medicine globally, there is an urgent need to formulate guidelines for the administration of Chinese medicine decoctions, which will provide a reference basis for informing patients of the administration of Chinese medicine decoctions when they are dispensing instructions.

Based on the systematic compilation of knowledge on the administration of Chinese herbal coction and with reference to the relevant laws and regulations of various countries and regions, this document develops a framework of knowledge system and specific guidance methods for the administration of Chinese medicine decoctions, with a view to promoting the standardization of the administration of Chinese medicine decoctions around the world. If the content of this document conflicts with the laws and regulations of the country where the standard is applied, the laws and regulations of that country will take precedence.

1 Scope

This document stipulates the basic principles, general guidance and personalized guidance on the administration of commonly used Chinese medicine decoctions.

This document applies to persons taking Chinese medicine decoctions, including patients and patients' family members; and professional instructors, including physicians, pharmacists, and dispensers.

2 Normative references

The contents of the following documents constitute indispensable provisions of this document through normative references in the text. Where a document is cited with a date, only the version corresponding to that date applies to this document; where a document is cited without a date, the latest version (including all change orders) applies to this document.

SCM 55-2020 *Requirements for Prescription, Dispensing, Dispensing and Decoction of Chinese Medicines Part 4: Requirements for Decoction of Chinese Medicines*

T/CACM 1366-2021 *Decocting Technical Specification for Traditional Chinese Medicine*

3 Terms and definitions

The following terms and definitions apply to this document.

3. 1

Chinese medicine decoction pieces

Chinese medicine decoction pieces refers to medicines that can be directly used in Chinese medicine clinics or preparation production for use after processing and concocting Chinese herbal medicines.

3. 2

Chinese medicine decoctions

Liquid dosage forms in which Chinese medicine decoction pieces are decocted with water, and the juice is removed from the dregs and taken for internal use; or liquid preparations such as Chinese herbal medicine formulated granules that are taken after brewing.

3. 3

Taking method of Chinese medicine decoctions

A scientific and rational approach to taking herbal decoction on time, in quantity, and according to the course of treatment.

4 Basic Principles of Taking Chinese medicine decoctions

4.1 According to the age, gender and severity of the disease, patients should follow the principle of "applying the dosage according to the evidence, taking the dosage to the extent of knowledge and stopping the medication after getting the effect" in taking Chinese medicine decoctions.

4.2 Guidance on taking Chinese medicine decoctions should include two parts, namely, general guidance and personalized guidance. Patients and their family members can refer to the content of general guidance, including taking time, frequency of dosage, taking temperature, dosage, course of treatment, storage guidelines, special taking method, and medication self-recording, with 8 parts, the framework of which can be found in Appendix A.1. Physicians, pharmacists, pharmacists and other professional practitioners should, based on general guidance, add personalized guidance content as appropriate, including warnings, contraindications to use and post-medication care in 3 parts, the framework see Appendix A.2. Guidance content should follow the following principles.

(a) The guidance for taking Chinese medicine decoctions should refer to the reference guidelines in Appendix B, and the knowledge should be extracted from the laws and regulations of various countries, classic teaching materials and practical experience.

b) The content of guidance on categorization of commonly used Chinese medicine decoctions is appropriate to refer to the categorization method given in Appendix C.

5 General guidance on taking Chinese medicine decoctions

5.1 Taking Time

5.1.1 The time of administration of Chinese medicine decoctions shall be in accordance with "7.1.2" and "7.1.3" of SCM 0055-2020.

5.1.2 Chinese medicine decoctions for the treatment of the upper-jiao in Chinese medicine (referring to the area above the chest and diaphragm, including the heart and lungs, etc.) should be taken 30 minutes after meals.

5.1.3 Chinese medicine decoctions for the treatment of the lower-jiao in Chinese medicine (referring to the area below the waist and abdomen, including the liver, kidneys, intestines, etc.) should be taken 30 to 60 minutes before meals.

5.1.4 Chinese medicine decoctions for the treatment of TCM li-syndrome (meaning that the disease manifests itself in the bone marrow) should be taken 30 minutes after dinner.

5.1.5 Chinese medicine decoctions for the treatment of TCM superficial syndrome(meaning that the disease manifests itself in the limbs or blood) should be taken within 8 to 10 hours without food and before eating the next morning.

5.1.6 Chinese medicine decoctions with the efficacy of antidieting or gastrointestinal stimulant is recommended to be taken 30 minutes after meal intake.

5.1.7 Chinese medicine decoctions with the efficacy of moving, dispersing and facilitating are

recommended to be taken 30~60 minutes before meals.

5.1.8 Chinese medicine decoctions with the effecacy of nourishing, cooling down and expelling water, attacking accumulation and directing stagnation, and expelling worms should be taken within 8 to 10 hours without food, and in the morning of the following day before eating.

5.1.9 Chinese medicine decoctions for the treatment of acute and serious illnesses may be taken at any time without following a specific dosage schedule or in accordance with medical advice.

5.1.10 Chinese medicine decoctions for the treatment of chronic diseases should be taken at regular intervals or as prescribed by a doctor.

5.2 Frequency of Dosage

5.2.1 The frequency of dosage of Chinese medicine decoctions shall be in accordance with SCM 0055-2020, "7.1 General Requirements".

5.2.2 Treatment of Chinese medicine diseases manifested as lung diseases, asthma, gastritis and other nighttime aggravation of the main, it is desirable to take a number of times at certain intervals, day and night consecutively.

5.2.3 Treatment of Chinese medicine diseases manifested wind-cold-dampness causing illness or frequent attacks, it is advisable to take a number of consecutive doses at certain intervals, day and night.

5.2.4 Treatment of Chinese medicine diseases manifested as acute or serious illnesses or predominantly upper-jiao illnesses, such as vomiting and pharyngeal diseases, it is advisable to take small amounts of the product several times.

5.2.5 Treatment of severe illness, poisoning and sudden death, 1 rapid dose is appropriate.

5.2.6 Treatment of acute, hyperthermia-type symptoms, one rapid dose is appropriate.

5.2.7 Chinese medicine decoctions which are harsh, toxic or have abortifacient effects should be taken once.

5.2.8 Chinese medicine decoctions with anthelmintic properties should be taken in one quick dose.

5.3 Taking temperature

5.3.1 The optimal temperature for Chinese medicine decoctions should be 30℃~37℃, and the maximum temperature should not exceed 65℃, or as prescribed by the doctor.

5.3.2 Treatment of Chinese medicine diseases manifested as hyperthermia, hemorrhagic fever, heatstroke, constipation, heat drenching, vomiting, poisoning, etc. It is advisable to take it cold and below 26℃.

5.3.3 Treatment of Chinese medicine diseases manifested as acute, gastrointestinal pain, internal cold syndrome, spleen and stomach deficiency cold syndrome, it is advisable to take warm, 37 ℃ ~ 40 ℃.

5.3.4 Treatment of Chinese medicine diseases manifested as true cold and false heat evidence, it is advisable to take cold and below 26℃;

5.3.5 Treatment of Chinese medicine diseases manifested as real heat and false cold evidence, it is advisable to take warm, 37 ℃ ~ 40 ℃.

5.3.6 Chinese medicine decoctions with the efficacy of tonic and offensive effects, it is advisable to take warm, 37℃~40℃.
5.3.7 Chinese medicine decoctions with the efficacy of stopping bleeding, astringency, removing heat, detoxification, stopping vomiting and clearing heat, it is advisable to take cold and below 26℃.
5.3.8 Chinese medicine decoctions with the efficacy of pungent warmth to relieve epidemics, regulate qi, invigorate blood circulation and remove blood stasis, dispel cold, and warm the blood circulation, it is advisable to take warm, 37℃~40℃.

5.4 Dosage

5.4.1 Daily dose to 1 day 1 dose is appropriate, the first decoction, the second decoction to merge, decoction out of the liquid 400 ~ 600mL, divided into 2 ~ 3 times; 200 ~ 300mL, divided into 1 time, or as directed by the doctor.
5.4.2 For adults, each dose should be 200-300mL; for infants less than 1 year old, each dose should be 1-5mL (the liquid should be concentrated); for children 1-2 years old, each dose should be 20-40mL (the liquid should be concentrated); for preschoolers 3-7 years old, each dose should be 40-80mL; for children 8-16 years old, each dose should be 100-150mL; in special cases or as directed by the physician. For children aged 8~16 years, the dose should be 100~150mL; in case of special circumstances, follow the doctor's instructions.

5.5 Course of Treatment

5.5.1 Relatively short courses of treatment for mild illnesses and relatively long courses of treatment for severe illnesses, as specified or prescribed by the physician.
5.5.2 Treatment of Chinese medicine diseases as acute and serious diseases, or with blood in urine, pain, diarrhea, etc. as the main manifestation, or with the efficacy of tranquilizing the spirit, activating the blood, sweating, etc., see the effect of stopping the drug, and comply with the doctor's instructions to carry out the medication.
5.5.3 Chinese medicine decoctions containing Pi Shi (red Pi Shi, white Pi Shi), Pi Shuang, Shui Yin, raw Ma Qian Zi, raw Chuan Wu, raw Cao Wu, raw Bai Fu Zi, raw Fu Zi, raw Ban Xia, raw Nan Xing, raw Ba Dou, Ban Mao, Qing Niang Zi, Hong Niang Zi, raw Gan Sui, raw Lang Du, raw Teng Huang, raw Qian Jin Zi, raw Tian Xian Zi, Nao Yao Hua, Xueshang Yizhihao, Red Sheng Dan, White Jiang Dan, Chan Su, Yang Jinhua, Hong Fen, Qing Fen, Xiong Huang, or other toxic Chinese medicine decoction pieces are included in the formula. Toxic varieties of Chinese medicines, take a course of treatment as short as possible, or follow the doctor's instruction.

5.6 Storage Guidelines

5.6.1 For all types of Chinese herbal pharmacies and clinics of traditional Chinese medicine temporary storage of decoction, should be based on the local or medical institutions; appropriate in the container boiling, after the bag packaging, in order to storage time is relatively long.
5.6.2 Vacuum-sealed packaged decoction should be placed in the refrigerator freezer within the range of 2 ℃ ~ 8 ℃ storage time within two weeks.

5.6.3 Chinese medicine decoctions should be stored at room temperature under ventilation, the storage time should not exceed 3 days, and should be re-boiled before consumption.
5.6.4 Chinese medicine decoctions containing animal-type Chinese medicine decoction pieces such as Quan Xie, Di Long, Wu Gong, etc., or with high sugar and high starch content such as ripened ripened Di Huang, Huang Qi, Ge Gen, etc., are recommended to be kept in refrigerator at low temperature for a period of not more than 7 days and should be re-boiled before consumption.
5.6.5 For decoctions decocted on behalf of medical institutions, if there are abnormal phenomena such as bulging of the medicine bag or discoloration, change of taste or bubbling of the medicine, they should not be consumed; for decoctions decocted on one's own, if discoloration, change of taste or other abnormal phenomena occur during the storage process, they should not be consumed.

5.7 Specialized Taking Methods

5.7.1 Chinese herbal medicines taken by "melting" shall comply with the provisions of "9.2.2.4 Melting (dissolving)" in T/CACM 1366-2021.
5.7.2 Chinese herbal medicines taken by "decocting separately" shall comply with the provisions of "9.2.2.3 Separate decoction (separate stew)" in T/CACM 1366-2021.
5.7.3 Chinese herbal medicines taken by "taking drenched" should be taken by combining the minced medicines with the prepared decoction and stirring.

5.8 Medication Self-Recording

5.8.1 Taking Chinese medicine decoctions with the effect of laxative or anthelmintic recording the time and frequency of defecation and the self-perception during defecation.
5.8.2 Taking Chinese medicine decoctions with the effect of inducing dampness and expelling water, and recording the color, odor, frequency of urination and the amount of urine.
5.8.3 Taking Chinese medicine decoctions that are strong in nature or contain poisonous pieces, observe the situation of vomiting and abdominal pain, and stop the medication immediately and seek medical advice if there is any toxic phenomenon such as severe abdominal pain, vomiting, profuse sweating, panic and shortness of breath.
5.8.4 Liver and kidney functions are monitored regularly, approximately every 3 months, when treatment is carried out with continuous use of herbal decoction.

6 Personalized guidance for taking Chinese medicine decoctions

6.1 Warnings

6.1.1 Prescription of Chinese medicine decoctions containing Pi Shi (red Pi Shi, white Pi Shi), Pi Shuang, Shui Yin, raw Ma Qian Zi, raw Chuan Wu, raw Cao Wu, raw Bai Fu Zi, raw Fu Zi, raw Ban Xia, raw Nan Xing, raw Ba Dou, Ban Mao, Qing Niang Zi, Hong Niang Zi, raw Gan Sui, raw Lang Du, raw Teng Huang, raw Qian Jin Zi, raw Tian Xian Zi, Nao Yao Hua, Xueshang Yizhihao,

Red Sheng Dan, White Jiang Dan, Chan Su, Yang Jinhua, Hong Fen, Qing Fen, Xiong Huang, etc. Toxic Chinese medicine varieties, need to indicate the content of the warning.
6.1.2 For additional information, see Appendix B, "Warnings".

6.2 Contraindications to Use

6.2.1 Drug-Drug

6.2.1.1 Contraindications associated with Chinese medicine decoctions shall be in accordance with SCM 0055-2020, Appendix C, "C.1 Contraindications to Compounding" and "C.2 Contraindications to Pregnancy".
6.2.1.2 When Chinese medicine decoctions are used in combination with other western medicines, the two medicines should be separated by 0.5 hours or more than 1 hour.
6.2.1.3 For additional information, see Appendix B, "Drug - Drug Contraindications" .

6.2.2 Drug-Population

6.2.2.1 Chinese medicine decoctions containing toxic Chinese medicine decoction pieces such as Pi Shi (red Pi Shi, white Pi Shi), Pi Shuang, Shui Yin, raw Ma Qian Zi, raw Chuan Wu, raw Cao Wu, raw Bai Fu Zi, raw Fu Zi, raw Ban Xia, raw Nan Xing, raw Ba Dou, Ban Mao, Qing Niang Zi, Hong Niang Zi, raw Gan Sui, raw Lang Du, raw Teng Huang, raw Qian Jin Zi, raw Tian Xian Zi, Nao Yao Hua, Xueshang Yizhihao, Red Sheng Dan, White Jiang Dan, Chan Su, Yang Jinhua, Hong Fen, Qing Fen, Xiong Huang, children, patients with liver and kidney insufficiency should be cautious, women in pregnancy is prohibited.
6.2.2.2 Chinese medicine decoctions with the effecacy of promoting menstruation and eliminating blood stasis, promoting qi circulation and breaking up stagnation, or Chinese medicine decoctions that are pungent, hot, dry and strong, or that are slippery to the passage of orifices, should be used with caution by women during pregnancy.
6.2.2.3 Chinese medicine decoctions with the efficacy of invigorating blood circulation and removing blood stasis, breaking blood circulation and opening up channels, promoting orifices, cathartic effect, expelling water and stopping bleeding should be used with caution for women during menstruation.
6.2.2.4 Older persons should not be given more than three to four drugs in combination with other drugs during treatment.
6.2.2.5 For the elderly, the dosage of Chinese herbal medicines should be 3/4 of the adult dosage for those aged 60-69 years, 3/5 of the adult dosage for those aged 70-79 years, 1/2 of the adult dosage for those aged 80-89 years, and the dosage for those aged 90 years and above should be modeled on that for children.
6.2.2.6 The abuse of tonic Chinese medicine decoctions is prohibited for the elderly.
6.2.2.7 Patients with hepatic or renal insufficiency avoid stacking drugs with similar effects.
6.2.2.8 For additional information, see Appendix B, “Drug-Population Contraindications” .

6.2.3 Drug-Disease

6.2.3.1 Avoid cold and raw food in patients with cold disorders; avoid pungent, hot and greasy food in patients with heat disorders.
6.2.3.2 Avoid deep-fried, cold, hard and indigestible foods for people with weak spleen and stomach and indigestion.
6.2.3.4 Patients with hyperactivity of Liver yang should avoid eating pepper, chili, garlic and drinking alcohol.
6.2.3.5 Patients with allergies should avoid eating hairy foods such as mango, lychee, peanuts and seafood.
6.2.3.6 Avoid seafood, mutton and other hairy products for patients with skin diseases.
6.2.3.7 Avoid pickled foods and salt in patients with oedematous diseases.

6.2.4 Drugs - Food/Health Supplements

6.2.4.1 General drug-food contraindications for Chinese medicine decoctions include avoidance of cold, spicy, greasy and irritating foods.
6.2.4.2 Avoid tea and alcohol during the administration of herbal decoction.
6.2.4.3 Avoid fatty meats and animal organs in patients with chest palsy.
6.2.4.4 Avoid acidic food and cold drinks for Chinese medicine decoctions with the effect of sweating and relieving exopathogens.
6.2.4.5 Chinese medicine decoctions with the effecacy of clearing heat and cooling Blood, detoxifying swelling, calming the Liver, moistening the Lungs, and brightening the eyes, and avoiding pungent and warm stimulants such as wine, garlic, coffee, chili, and mutton.
6.2.4.6 Chinese medicine decoctions with the effecacy of warming menstruation, tonifying yang, astringing essence and stopping diarrhea, dispelling wind-dampness and relieving cold and pain, avoid cold drinks, raw pears, crabs, persimmons, bamboo shoots and other cold products.
6.2.4.7 Foods made of glutinous rice are contraindicated for herbal decoction with stomach nourishing properties.
6.2.4.8 Sugar should not be added to decoctions with the efficacy of expelling water, diuretic, diuretic, etc. Sugar should not be added to Chinese medicine decoctions with warm and hot properties, and brown sugar should not be added to Chinese medicine decoctions with cold and cool properties.
6.2.4.9 Ban Xia Fu Ling Decoction is used to treat malignant obstruction of pregnancy, avoiding cold, vinegar, slippery grease, woad, and seaweed; and Du Huo Decoction is used to treat malignant wind and poisonous qi, avoiding gourd and houttuynia cordata.
6.2.4.10 Gan Cao Decoction for migraine for years without disease be cautious of wind and cold, vinegar, garlic, noodles, milk, cheese, and fish in the course of taking the medicine.

6.2.5 Drug-Environment

6.2.5.1 Chinese medicine decoctions with the effecacy of tranquilizing the fetus should be taken in a warm position and the room environment should be kept quiet.
6.2.5.2 Chinese medicine decoctions with efficacy of dispelling wind-dampness should not be taken in a humid environment or underwater work should be avoided.
6.2.5.3 Chinese medicine decoctions for treating wind-induced illnesses need to be protected from wind after administration.
6.2.5.4 For additional information, see Appendix B, "Drug-Environmental Contraindications".

6.3 Post-Medication Care

6.3.1 Nursing Care

6.3.1 For decoctions that require "sipping hot porridge" to assist the drug effect, it is advisable to take a small amount of hot porridge (above 60°C) after taking the drug.
6.3.2 For decoctions that require "warm covers" to assist the medicinal effects, it is advisable to lie down under a blanket after taking the medication.
6.3.3 For decoctions that require "stopped at the time of effect," etc., the medication should be stopped immediately if there is an improvement in indicators and symptoms.
6.3.4 Chinese medicine decoctions with efficacy of vomiting, it is advisable to take ginger juice, cold thin porridge (30°C is appropriate) or cool boiled water to stop vomiting.
6.3.5 Chinese medicine decoctions with efficacy of tranquilizing the fetus, it is advisable to lie down with appropriate warmth after taking the medicine.
6.3.6 Chinese medicine decoctions for treating gastrointestinal disorders, it is advisable to lie down appropriately warm after taking the medicine.

6.3.2 Possible Adverse Reactions

6.3.2.1 Toxic reactions such as dizziness, nausea, and toxic shock may occur when Chinese medicine decoctions are taken inadvertently or in excessive doses, and the medication should be stopped immediately and resuscitation carried out.
6.3.2.2 When taking Chinese medicine decoctions containing Chinese herbal medicinal pieces with alkaloid ingredients, manifestations of poisoning related to the central nervous system and the autonomic nervous system, such as slurred speech, nausea and vomiting, respiratory distress, and cardiac arrhythmia, may occur after taking the medication.
6.3.2.3 When taking Chinese medicine decoctions containing Chinese herbal medicinal pieces with toxic proteinaceous ingredients, manifestations of intoxication related to digestive and neurological dysfunction, such as erythrocytolysis and generalized widespread hemorrhage, may occur after taking the medication.
6.3.2.4 When taking Chinese medicine decoctions containing toxic glycosides of Chinese medicine decoction pieces may have central nervous system and cardiac related toxic manifestations such as blood in stool, fainting, numbness of limbs, respiratory arrest, cardiogenic

cerebral insufficiency of blood supply, etc., after taking the medication.

6.3.2.5 When taking herbal decoction containing terpenoids and lactones, gastrointestinal and liver-related toxicity manifestations, such as generalized numbness, chest tightness and severe vomiting, may occur after administration.

6.3.2.6 When taking Chinese medicine decoctions containing animal Chinese medicine decoction pieces, liver, kidney and cardiovascular related toxicity manifestations, such as blood in stool, paralysis of both lower limbs, difficulty in urination and defecation, vomiting of blood, etc., may occur after taking the medication.

6.3.2.7 When taking Chinese medicine decoctions containing mineral-based Chinese herbal medicinal tablets, manifestations of poisoning related to the nervous system, hematopoietic system, digestive system, cardiovascular system, as well as the liver and kidneys, such as respiratory distress, dizziness and dizziness, and locomotive spasm, etc., may occur after taking the medicine.

6.3.2.8 For additional information, see Appendix B, "Possible Adverse Reactions" .

6.3.3 Effects on Excretory Production After Drug Administration

Taking Chinese medicine decoctions containing Chinese medicine decoction pieces including Zhi Zi, Dan Shen, Niu Huang, Da Huang, Fan Xie Ye, Lu Hui, Huang Lian, Huang Qin may cause urine color changes in accordance with Table 1 and symptoms can disappear spontaneously after stopping the decoctions; If you feel uncomfortable, stop taking the decoctions immediately and seek medical attention.

Table 1 Possible effects on excretion after drug administration

Chinese medicine decoction pieces	Effects on urine color
Zhi Zi, Dan Shen	Red
Niu Huang	Yellow
Da Huang, Fan Xie Ye, Lu Hui	Deep Yellow
Huang Lian, Huang Qin	Yellowish Green

6.3.4 Medication Monitoring

6.3.4.1 Chinese medicine decoctions with efficacy of laxative or anthelmintic, the number of stools, quality, color, odor, and whether worms are eliminated should be monitored; if the number and quantity of stools are high, the medication may be discontinued; if there is no bowel movement for 3-4 hours after taking the medication, the dose of the medication may be increased.

6.3.4.2 Chinese medicine decoctions with efficacy of diuretic and water-eliminating, the color, odor, frequency of urine and the amount of urine should be monitored, and the medication may be discontinued if the frequency and amount of urine are high.

6.3.4.3 Chinese medicine decoctions that are very strong or contain toxic drinks should be monitored for blood pressure, vomiting, and abdominal pain.

Appendix A
(normative)
Knowledge Framework for Guidelines on Taking Chinese medicine decoctions

At the time of dispensing, the pharmacist's guidance to the patient on the taking of Chinese medicine decoctions should comprise two parts, namely, generic guidance and personalized guidance, and the guidance should be tailored according to the patient's gender, age, physique, the location, condition and mechanism of the disease and the severity of the disease, with a clear distinction between the primary and secondary parts. The guidance for patients can be seen in Figure A.1, and the guidance for physicians/pharmacists and other professional practitioners can be seen in Figure A.2.

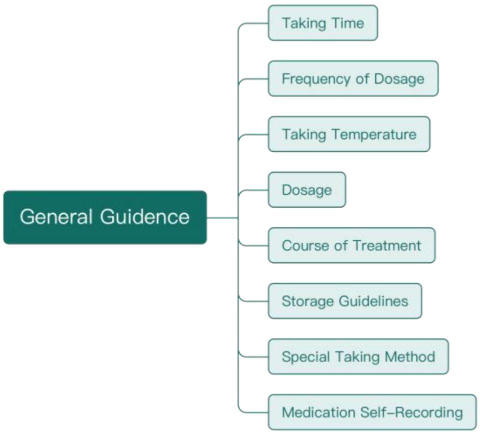

Figure A.1 Knowledge framework for general guidance on Chinese medicine decoctions

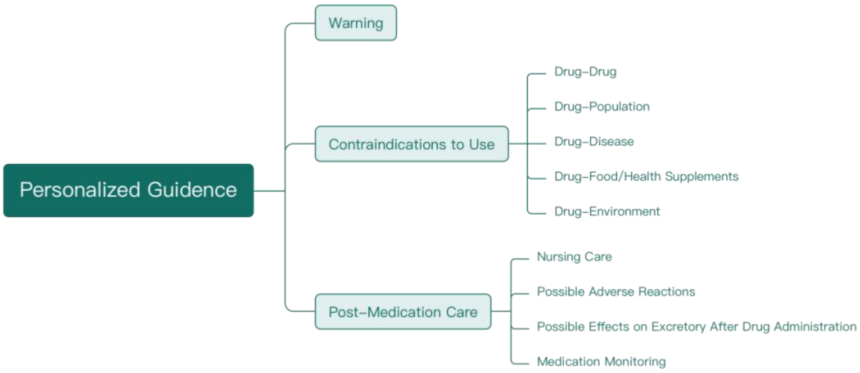

Figure A.2 Knowledge framework for personalized guidance on Chinese medicine decoctions

Appendix B
（Informative）

Reference Guidelines for the Knowledge Framework for the Guidelines for the Administration of Traditional Chinese Medicinal Decoction

The framework of the knowledge system for taking Chinese medicine decoctions includes 7 level 1 knowledge entries such as "Names and basic therapeutic effects of Chinese medicine decoctions", 13 level 2 knowledge entries such as "Duration of taking Chinese medicine decoctions" and 82 reference guidelines such as "Chinese Pharmacopoeia (2020 Edition)". 2020 Edition)" and 82 reference guidelines. Under the knowledge system of guidelines for taking Chinese medicine decoctions, 82 knowledge contents can be extracted with reference to existing Chinese medicine regulations, standards, teaching materials and other documents (see Table B.1).

Table B.1 Two Levels of Elements of Guidelines for the Administration of TCM decoction and Their Reference Guidelines

<table>
<tr><th>First-Level Elements</th><th>Second-Level Elements</th><th>Reference Guidelines</th><th>Knowledge content number</th></tr>
<tr><td rowspan="2">1 Name and Basic Effects</td><td rowspan="2">——</td><td>1-Ancient Chinese Medical Texts "Treatise on Typhoid Miscellaneous Diseases" "Bijie Qianjin Yaofang</td><td>1</td></tr>
<tr><td>2-Formulary (Ji Li, Zhengyun Zuo, eds., China TCM Press, 2021).</td><td>2</td></tr>
<tr><td rowspan="9">2 Dosage (including information on special dosage methods for TCM decocting pieces, such as Yanghua, etc.)</td><td rowspan="3">1. Taking Time</td><td>1-Chinese Medical Dictionary (5th edition)</td><td>3</td></tr>
<tr><td>2-A Guide to the Diagnosis and Treatment of Internal Common Diseases in Traditional Chinese Medicine</td><td>4</td></tr>
<tr><td>3-Formulary (Ji Li, Zhengyun Zuo, eds., China TCM Press, 2021).</td><td>5</td></tr>
<tr><td rowspan="4">2. Frequency of Dosage</td><td>1-Formulary (Ji Li, Zhengyun Zuo, eds., China TCM Press, 2021).</td><td>6</td></tr>
<tr><td>2-A Guide to the Diagnosis and Treatment of Internal Common Diseases in Traditional Chinese Medicine</td><td>7</td></tr>
<tr><td>3-Formulary (Ji Li, Zhengyun Zuo, eds., China TCM Press, 2021).</td><td>8</td></tr>
<tr><td>4-Clinical randomized controlled trials</td><td>9</td></tr>
<tr><td rowspan="2">3.Taking Temperature</td><td>1-Chinese Medical Dictionary (5th edition)</td><td>10</td></tr>
<tr><td>2-A Guide to the Diagnosis and Treatment of Internal Common Diseases in Traditional</td><td>11</td></tr>
</table>

		Chinese Medicine	
		3-Formulary (Ji Li, Zhengyun Zuo, eds., China TCM Press, 2021).	12
	4.Dosage	1-Chinese Medical Dictionary (5th edition)	13
		2-A Guide to the Diagnosis and Treatment of Internal Common Diseases in Traditional Chinese Medicine	14
		3-Formulary (Ji Li, Zhengyun Zuo, eds., China TCM Press, 2021).	15
		4-Clinical randomized controlled trials	16
	5.Course of Treatment(advise if available)	1-Chinese Medical Dictionary (5th edition)	17
		2-A Guide to the Diagnosis and Treatment of Internal Common Diseases in Traditional Chinese Medicine	18
		3-Formulary (Ji Li, Zhengyun Zuo, eds., China TCM Press, 2021).	19
		4-Clinical randomized controlled trials	20
Storage Guidelines of TCM decoction	——	1-Practical clinical experience with medications (depending on geography and healthcare organization)	21
Warning	——	1-Chinese Pharmacopoeia, 2020 Edition	22
		2-The Chinese Medicine Volume of the Pharmacopoeia of the People's Republic of China for Clinical Use (National Pharmacopoeia Committee, People's Health Publishing House, 2020).	23
		3-Guide to the Clinical Application of National Essential Drugs (Editorial Committee of the Guide to the Clinical Application of National Essential Drugs, People's Health Publishing House, 2010).	24
		4-Domestic and foreign periodical databases	25
Contraindications for taking TCM decoction	1.Drug-Drug (including Chinese-Western drug combinations)	1-Adverse Drug Reaction Monitoring Database	26
		2-Chinese Pharmacopoeia, 2020 Edition)	27
		3-The Chinese Medicine Volume of the Pharmacopoeia of the People's Republic of China for Clinical Use (National Pharmacopoeia Committee, People's Health Publishing House, 2011).	28
		4-Preclinical studies, pre-market clinical trials and post-market re-evaluation studies collected by marketing authorization holders of pharmaceutical products	29

		5-Clinical Chinese Medicine Pharmacotherapy (Bing Zhang, Zhenxiang Zhou, eds., People's Health Publishing House, 2016).	30
		6-Domestic and foreign periodical databases	31
		7-Chinese Medical Dictionary (5th edition)	32
		8-Interactions between Proprietary Chinese Medicines and Western Medicines (Cao Junling, Zhen Hanshen, eds., People's Health Publishing House, 2016)	33
	2.Drug-Population	1-Adverse Drug Reaction Monitoring Database	34
		2-The Chinese Medicine Volume of the Pharmacopoeia of the People's Republic of China for Clinical Use (National Pharmacopoeia Committee, People's Health Publishing House, 2011).	35
		3-Clinical Chinese Medicine Pharmacotherapy (Bing Zhang, Zhenxiang Zhou, eds., People's Health Publishing House, 2016).	36
		4-Domestic and foreign periodical databases	37
		5-Chinese Medical Dictionary (5th edition)	38
		6-Practical clinical experience with medication	39
	3.Drug-Food/Health Supplements	1-Adverse Drug Reaction Monitoring Database	40
		2-Chinese Pharmacopoeia, 2020 Edition)	41
		3-The Chinese Medicine Volume of the Pharmacopoeia of the People's Republic of China for Clinical Use (National Pharmacopoeia Committee, People's Health Publishing House, 2011).	42
		4-Preclinical studies, pre-market clinical trials and post-market re-evaluation studies collected by marketing authorization holders of pharmaceutical products	43
		5-Clinical Chinese Medicine Pharmacotherapy (Bing Zhang, Zhenxiang Zhou, eds., People's Health Publishing House, 2016).	44
		6-Domestic and foreign periodical databases	45
		7-Chinese Medical Dictionary (5th edition)	46
	4.Drug-Diseases	1-Adverse Drug Reaction Monitoring Database	47
		2-Chinese Pharmacopoeia, 2020 Edition)	48
		3-The Chinese Medicine Volume of the Pharmacopoeia of the People's Republic of China for Clinical Use (National Pharmacopoeia Committee, People's Health Publishing House, 2011).	49

		4-Preclinical studies, pre-market clinical trials and post-market re-evaluation studies collected by marketing authorization holders of pharmaceutical products	50
		5-Clinical Chinese Medicine Pharmacotherapy (Bing Zhang, Zhenxiang Zhou, eds., People's Health Publishing House, 2016).	51
		6-Domestic and foreign periodical databases	52
		7-Chinese Medical Dictionary (5th edition)	53
	5.Drug-Environment	1-Adverse Drug Reaction Monitoring Database	54
		2-Chinese Pharmacopoeia, 2020 Edition)	55
		3-The Chinese Medicine Volume of the Pharmacopoeia of the People's Republic of China for Clinical Use (National Pharmacopoeia Committee, People's Health Publishing House, 2011).	56
		4-Preclinical studies, pre-market clinical trials and post-market re-evaluation studies collected by marketing authorization holders of pharmaceutical products	57
		5-Clinical Chinese Medicine Pharmacotherapy (Bing Zhang, Zhenxiang Zhou, eds., People's Health Publishing House, 2016)	58
		6-Domestic and foreign periodical databases	59
		7-Chinese Pharmacopoeia, 2020 Edition)	60
Post-medication care of TCM decoction	1.Nursing Care	1-Chinese Medical Dictionary (5th edition)	61
		2-A Guide to the Diagnosis and Treatment of Internal Common Diseases in Traditional Chinese Medicine	62
		3-Pharmacovigilance in Traditional Chinese Medicine (Bing Zhang, ed., People's Health Publishing House, 2015)	63
		4-Internal Medicine of TCM (Mianhua Wu, Yan Shi, eds., China Press of Traditional Chinese Medicine, 2021).	64
		5-The Chinese Medicine Volume of the Pharmacopoeia of the People's Republic of China for Clinical Use (National Pharmacopoeia Committee, People's Health Publishing House, 2011).	65
		6-Domestic and foreign periodical databases	66
		7-Preclinical studies, pre-market clinical trials and post-market re-evaluation studies collected by marketing authorization holders of	67

		pharmaceutical products	
		8-Clinical Chinese Medicine Pharmacotherapy (Bing Zhang, Zhenxiang Zhou, eds., People's Health Publishing House, 2016)	68
		9-Practical clinical experience with medication	69
	2.Preventive/Treatment Measures for Adverse Reactions (Including possible adverse reactions)	1-Chinese Medical Dictionary (5th edition)	70
		2-A Guide to the Diagnosis and Treatment of Internal Common Diseases in Traditional Chinese Medicine	71
		3-Pharmacovigilance in Traditional Chinese Medicine (Bing Zhang, ed., People's Health Publishing House, 2015)	72
		4-The Chinese Medicine Volume of the Pharmacopoeia of the People's Republic of China for Clinical Use (National Pharmacopoeia Committee, People's Health Publishing House, 2011).	73
		5-Preclinical studies, pre-market clinical trials and post-market re-evaluation studies collected by marketing authorization holders of pharmaceutical products	74
		6-Domestic and foreign periodical databases	75
		7-Clinical Chinese Medicine Pharmacotherapy (Bing Zhang, Zhenxiang Zhou, eds., People's Health Publishing House, 2016)	76
		8-Practical clinical experience with medication	77
	3.Medication Monitoring/Self-Recording	1-Practical clinical experience with medication	78
		2-Domestic and foreign periodical databases	79
	4.Possible Effects on Excretory after Drug Administration	1-Chinese Medical Dictionary (5th edition)	80
		2-Practical clinical experience with medication	81
		3-Pharmacovigilance in Traditional Chinese Medicine (Bing Zhang, ed., People's Health Publishing House, 2015)	82

Appendix C
(Informative)

Catalog of Commonly Used Chinese medicine decoctions

C.1 Index of Examples of Commonly Used TCM Decoction

This part of the content in accordance with the disease classification method of the State Administration of Traditional Chinese Medicine released in 2018, "Ancient Classical Famous Prescription Catalog (the first batch)", the Eastern Han Dynasty - Zhongjing Zhang's "Treatise on Miscellaneous Diseases of Typhoid Fever", the Tang Dynasty - Simiao Sun's "Beijie Qianjin Yaofang" of the TCM decoction prescriptions for indexing and research, screening of those who have clear records of the name of the formula, composition of the prescription, main diseases, medication temperature, medication time, medication frequency, medication dosage, duration of the treatment, contraindications to the medicine, post-medication care, and observation of the therapeutic effect of the corresponding part of the record. And the prescriptions did not contain highly toxic Chinese medicines included in the Pharmacopoeia of the People's Republic of China (2020 edition), and did not involve the first-grade protected species in the catalog of wildlife medicinal herb species under national key protection; the prescriptions were commonly used Chinese medicines or TCM decocting pieces prescriptions, which did not contain the eighteen anti-toxicity, nineteen dangers and other contraindications for compounding. A total of 461 examples of commonly used Chinese medicine decoctions were screened.

**Classification of diseases**	**Types**		**Commonly Used Prescriptions**
Diseases of the Lungs	Cough	Cough of various qi	Houpo Decoction
		Palpitation	Jupi Decocton
		Lung Qi Deficiency	Bufei Decoction
		Lung Qi Deficiency,Lumbago	Xiaojianzhong Decoction
		Cough	Guiling Wuwei Gancao Decoction
			Linggan Wuwei Jiangxin Decoction
			Guiling Wuwei Gancao Quguijiajiangxinbanxia Decoction
			Jiajiangxinbanxia Xingren Decoction Prescription
			Qinganwuwei jiajiangxinbanxingdahuang Decoction
			Xiaoqinglong jia Shigao Decoction

			Shegan Mahuang Decoction
			Houpo Mahuang Decoction
			Maimendong Decoction
			Mahuang Shigao Decoction
			Shizao Decoction
			Wenpi Decoction
			Baibugen Decoction
			Haizao Decoction
			Baiqian Decoction
			Zhupi Decoction
	Lung Abscess	——	Jiegeng Decoction
			Xiefei Decoction
	Lung Consumption	Severe Lung Qi Deficiency	Mahuangyinqi Decoction
			Banxia Decoction
			Houpo Decoction
			Huangqi Decoction
			Daqianhu Decoction
			Zhuye Decoction
			Guizhiqushaoyao jiazaojia Decoction
	Children's Fetish Knot Distension and Fullness	——	Ziwan Decoction
			Wuweizi Decoction
			Shegan Decoction
			Guizhi Decoction
	Pulmonary Impotence	——	Gancao ganjiang Decoction
			Gancao Decoction
	Pediatric Typhoid Fever	——	Maimendong Decoction
			Shaoyaosiwujieji Decoction
			Mahuang Decoction
			Wuweizi Decoction
			Shengma Decoction
			Dahuang Decoction
			Zhuye Decoction
			Tiaozhong Decoction
			Shengdihuang Decoction
			Hengshan Decoction
	Cholera		Guizhi Decoction
			Guizhi plus gui Decoction
			Guizhi plus shaoyao Decoction
			Guizhi plus dahuang Decoction
			Guizhi plus houpoxingzi Decoction
			Guizhi plus fuzi Decoction

			Guizhi Decoction
			Guizhi reduce Shaoyao plus Fuzi Decoction
			Guizhi Gancao Longgu Muli Decoction
			Guizhi Renshen Decoction
			Guizhi Fuzi Decoction
			Guizhi reduce Gui plus Fulingbaizhu Decoction
			Guizhi plus Shaoyaoshengjiang geyiliang Renshensanliangxinjia Decoction
			Guizhi Mahuanggeban Decoction
			Mahuang Decoction
			Daqinglong Decoction
			Xiaoqinglong Decoction
			Guizhier Mahuangyi Decoction
			Guizhier Yuebiyi Decoction
			Mahunag Xixin Fuzi Decoction
			Mahuang Fuzi Gancao Decoction
			Mahuang Xingren Gancao Shigao Decoction
			Gegen Decoction
			Gegen plus Banxia Decoction
			Gegen Huangqin Huanglian Decoction
			Guizhi plus Gegen Decoction
			Baihu Decoction
			Baihu plus Renshen Decoction
			Zhuye Shigao Decoction
			Tiaowei Chengqi Decoction
			Xizochengqi Decoction
			Dachengqi Decoction
			Taohe chengqi Decoction
			Daxianxiong Decoction
			Xiaoxianxiong Decoction
			Zhizichi Decoction
			Zhizi Gancao Chi Decoction
			Zhizi Shengjiang Chi

			Decoction
			Zhizi Houpo Decoction
			Zhizi Ganjiang Decoction
			Zhizi Baipi Decoction
			Yinchenhao Decoction
			Zhishi Zhizichi Decoction
			Xiaochaihu Decoction
			Dachaihu Decoction
			Chaihu plus Mangxiao Decoction
			Chaihu Guizhi Decoction
			Chaihu Guizhi Ganjiang Decoction
			Huangqin Decoction
			Huangqin plus Banxia Shengjiang Decoction
			Dahuang Huanglian Xiexin Decoction
			Fuzi Xiexin Decoction
			Gancao Xiexin Decoction
			Banxia Xiexin Decoction
			Shengjiang Xiexin Decoction
			Ganjiang Huangqin Huanglian Renshen Decoction
			Huanglian Decoction
			Xuanfu Daizhe Decoction
			Sini Decoction
			Tongmai Sini Decoction
			Tongmai Sini plus Zhudanzhi Decoction
			Ganjiang Fuzi Decoction
			Sini plus Renshen Decoction
			Fuling Sini Decoction
			Fuzi Decoction
			Zhenwu Decoction
			Baitong Decoction
			Danggui Sini Decoction
			Dangguisini plus Wuzhuyu Shengjiang Decoction
			Gancao Decoction
			Jiegeng Decoction
			Guizhi Gancao Decoction
			Gancao Ganjiang Decoction

			Shaoyao Gancao Decoction
			Shaoyao Gancao Fuzi Decoction
			Fuling Gancao Decoction
			Fuling Guizhi Gancao Dazao Decoction
			Fuling Guizhi Baizhu Gancao Decoction
			Houpo Shengjiang Banxia Gancao Renshen Decoction
			Xiaojianzhong Decoction
			Zhigancao Decoction
			Chishizhi Yuyuliang Decoction
			Taohua Decoction
			Zhuling Decoction
			Wuzhuyu Decoction
			Mahuang Shengma Decoction
			Gancao Fuzi Decoction
			Baitouweng Decoction
			Guizhi Fuzi reduce gui plus Baizhu Decoction
			Guizhi Shaoyao Zhimu Decoction
			Huangqi Guizhi Wuwu Decoction
			Banxia Houpo Decoction
			Gualou Xiebai Banxia Decoction
			Lingguizhugan Decoction
			Zexie Decoction
			Baihedihuang Decoction
			Zhishi Xiebai Guizhi Decoction
			Jupi Zhuru Decoction
			Maimendong Decoction
			Ganjianglingzhu Decoction
			Houpo Qiwu Decoction
			Houpo Mahuang Decoction
			Danggui Jianzhong Decoction
			Wenpi Decoction
			Wendan Decoction
			Xiaoxuming Decoction
			Sanhua Decoction

Disease of the Heart	Tachycardia	Pregnancy Vexation(irritability and palpitations in mid-pregnancy)	Zhuli Decoction
		Palpitations of Weak Heart Qi	Dabuxin Decoction
		Shaking in Fear	Yuanzhi Decoction
			Fushen Decoction
			Buxin Decoction
			Xiaodingxin Decoction
			Dadingxing Decoction
			Jingli Decoction
			Zhenxin Decoction
	Chest Impediment	Solid heat in the heart with boredom	Shigao Decoction
		Constipation in the lower regions of the heart	Xiexin Decoction
		Heart pain	Guixinsanwu Decoction
			Wuxin Decoction
			Gaoliangjiang Decoction
			Wenzhongdanggui Decoction
			Yangroudanggui Decoction
			Wenpi Decoction
		Chest Impediment	Gualou Decoction
			Zhishi Xiebai Guizhi Decoction
			Fuling Decoction
			Tongqi Decoction
			Qianhu Decoction
			Binglang Decoction
	Heart-Laore	——	Dahuangxiere Decoction
			Shengma Decoction
			Mahuangtiaoxinxiere Decoction
	Other Diseases	Heart Qi Deficiency, hematemesis	Dahuanghuanglianxiexin Decoction
		Dreams, joy, laughter, fear, palpitations, restlessness.	Zhuli Decoction
		Heart Qi Deficiency	Fulingbuxin Decoction
		Heart deficiency and cold, heart swelling	Banxiabuxin Decoction

		and fullness	
Brain Disease	Dazzling	Dazzling	Fangfeng Decoction
	Stroke	Postpartum Stroke	Dadouzi Decoction
			Duhuozi Decoction
			Xiaoduhuo Decoction
			Gancao Decoction
			Duhuo Decoction
			Zhuye Decoction
			Fangfeng Decoction
			Duhuo Decoction
			Dadou Decoction
			Wushi Decoction
			Sishi Decoction
			Xiaochaihu Decoction
			Gancao Decoction
			Gegen Decoction
			Fushen Decoction
			Yuanzhi Decoction
			Fuling Decoction
			Anxin Decoction
		Stroke	Xiaoxuming Decoction
			Daxuming Decoction
			Xizhouxuming Decoction
			Paifeng Decoction
			Dabafeng Decoction
			Wutou Decoction
			Dafangfeng Decoction
		Thief Wind Stroke	Chuanxiong Decoction
			Yiyuanmahuangxuming Decoction
			Wenzhongshengjiang Decoction
			Wutou Decoction
			Fangfeng Decoction
			Qianghuo Decoction
			Fangji Decoction
			Dazao Decoction
		Contrary Wind	Fangfeng Decoction
			Gegen Decoction
			Zhongjingsanhuang Decoction
			Bailianyiyi Decoction
			Duhuojisheng Decoction
		Post-stroke Hemiplegia	Zhuli Decoction

		Severe stroke coma	Duhuo Decoction
		Opitoshtonous	Canggongdanggui Decoction
		Cardiac Diseases - Head and Face Wind	Fushen Decoction
			Xijiao Decoction
	Epilepsy	Epilepsy	Dahuang Decoction
			Zengsunxuming Decoction
			Shigao Decoction
Diseases of the spleen and stomach system	Vomiting	Obstruction of Pregnancy	Banxia Fuling Decoction
			Qingzhuru Decoction
			Jupi Decoction
		The stomach does not accept food and vomits when it is eaten	Dabanxia Decoction
		Vomiting	Banxia Decoction
			Qianhu Decoction
			Xiaomai Decoction
			Jupi Decoction
			Dahuanggancao Decoction
	Choke	Choke	Zhupi Decoction
			Ganjiang Decoction
			Tongqi Decoction
	Abdominal Pain	Postpartum Abdominal Pain	Shaoyao Decoction
			Danggui Decoction
			Yangrou Decoction
			Yangroudanggui Decoction
			Yangrouduzhong Decoction
			Yangroudihuang Decoction
			Neibudangguijianzhong Decoction
			Neibuchuanxiong Decoction
			Dabuzhongdanggui Decoction
			Wuzhuyu Decoction
			Puhuang Decoction
			Baijiang Decoction
			Duhuo Decoction
			Shaoyaohuangqi Decoction
	Loose Bowels	Postpartum Dysentery	Baitouweng Decoction
			Biejia Decoction
			Gandihuang Decoction
			Shengdihuang Decoction
	Typhoid	Dysentery characterized by heat	Sanhuangbaitouweng Decoction
			Baitouweng Decoction

			Fuling Decoction
		Dysentery	Wenpi Decoction
			Dataohua Decoction
			Cangmi Decoction
			Fuzi Decoction
			Duanli Decoction
			Xiexin Decoction
			Xiangsu Decoction
		Pediatric Dysentery	Wenzhong Decoction
			Wenzhong Dahuang Decoction
			Dahuang Decoction
	Insomnia	——	Sanhuang Decoction
	Other Diseases	Jaundice due to spleen fever	Dahuangxiere Decoction
		Depletion of the spleen	Banxia Decoction
		Bitterness in the mouth	Buwei Decoction
		Bloating	Wenwei Decoction
			Dabanxia Decoction
			Fuzi Jingmi Decoction
			Houposanwu Decoction
			Houpoqiwu Decoction
			Wuzhuyu Decoction
			Dagui Decoction
		Chronic cold and heat	Banxia Decoction
			Shengjiang Decoction
			Gancao Decoction
			Zhuyu Xiaoshi Decoction
			Dahuang Fuzi Decoction
			Dawutou Decoction
			Wushi Decoction
			Zhuye Decoction
			Banxia Decoction
			Chengqi Decoction
disease in the liver and gallbladder system	Duress	Post-partum dystocia	Gandihuang Decoction
		Liver qi deficiency, dystocia	Bugan Decoction
		Liver deficiency and cold, distension and dystocia	Binglang Decoction
	Aggregation	Accumulation of hardened mucus	Xianxiong Decoction
			Gansui Decoction
	Other Diseases	Pediatric carbuncle and scrofula	Loulu Decoction
			Mahuang Decoction

		Cramps	Jupitongqi Decoction
Renal Diseases	Edema	Edema in pregnancy, dysuria	Liyu Decoction
	Other diseases	Postnatal Gonorrhea	Gualou Decoction
			Zhuye Decoction
		Abdominal distension and fullness with solid heat in the kidneys	Xieshen Decoction
		Excess heat in the kidneys caused by internal heat	Zhizi Decoction
		Essential qi Deficiency	Zhuyehuangqin Decoction
		Guji	Sanhuang Decoction
		Lumbago	Shenzhuo Decoction
Diseases of Qi,Blood,Fluid and Humor	Hematemesis	——	Shengdihuang Decoction
			Jianzhong Decoction
			Baiye Decoction
			Zelan Decoction
			Xijiaodihuang Decoction
			Danggui Decoction
			Zhuru Decoction
			Maimendong Decoction
	Phlegm-retained Fluid	——	Xiaobanxia Decoction
			Gancao Decoction
			Mufangji Decoction
			Houpo Dahuang Decoction
			Xiaobanxia plus Fuling Decoction
			Gansuibanxia Decoction
			Daguling Decoction
			Fuling Decoction
			Dabanxia Decoction
			Banxia Decoction
			Danggui Decoction
			Wuzhuyu Decoction
			Qianhu Decoction
			Xuanfuhua Decoction
			Jiangjiao Decoction
			Duange Decoction
	Consumptive Disease	Postpartum Consumptive Disease	Yangrou Decoction
			Zhushen Decoction
			Yaorouhuangqi Decoction
			Dangguishaoyao Decoction

			Xingren Decoction
			Wushi Decoction
			Sanshi Decoction
			Neibu Huangqi Decoction
			Wuzhuyu Decoction
			Liyu Decoction
			Guizhi plus Fuzi Decoction
		Postpartum Vexation of Deficiency Type	Xiebai Decoction
			Renshen Danggui Decoction
			Ganzhuru Decoction
			Zhimu Decoction
			Zhuye Decoction
			Danzhuru Decoction
			Shaoyao Decoction
		Gallbladder Qi Deficiency leads to Vexation of Deficiency Type	Wendan Decoction
			Suanzao Decoction
			Zhizi Decoction
		Consumptive Disease	Jianzhong Decoction
			Xiaojianzhong Decoction
			Huangqinjianzhong Decoction
			Qianhujianzhong Decoction
			Lelingjianzhong Decoction
			Huangqin Decoction
			Dajianzhong Decoction
			Shenli Decoction
			Zengsunshenli Decoction
			Ningtuo Decoction
			Renshen Decoction
Disease in the limbs and meridians	Paralysis	Common cold sore	Diyizhuli Decoction
			Disanzhuli Decoction
			Mahuang Decoction
			Dierfuduhuo Decoction
			Disifufuhuo Decoction Prescription
			Duhuo Decoction
			Yuebi Decoction
			Fengyin Decoction
			XIjiao Xuanfuhua DecoctionPrescription
			Daxijiao Decoction
			Wuzhuyu Decoction
			Xiaofengyin Decoction

			Siwu Fuzi Decoction
			Banxia Decoction
			Zisuzi Decoction
			Fuzi Decoction
			Fangfeng Decoction
			Gancao Decoction
	Atrophy	——	Jiefengbi Decoction
			Xizhouxuming Decoction
Diseases Of Seven Orifices	Eye disease	Be Unable to See Clearly	Xiegan Decoction
	Nose disease	Nosebleed	Shengdihuang Decoction
Other diseases	——	Sterility	Piaoxiaodangbao Decoction
		Prevent Abortion	Wuciji Decoction Prescription
			Butai Decoction
			Aiye Decoction Prescription
			Huanglian Decoction
			Xiongji Decoction Prescription
			Fushen Decoction
			Juhua Decoction Prescription
			Anzhong Decoction Prescription
			Maimendong Decoction Prescription
			Chaihu Decoction
			Congbai Decoction Prescription
			Xingren Decoction
			Shaoyao Decoction
			Banxia Decoction
			Zhushen Prescription
		Threatened Abortion	Congbai Decoction
			Xuanfuhua Decoction
			Jiaoai Decoction
		Promote lactation	Danxingguijian Decoction
		Lochia	Gandihuang Decoction
			Zelan Decoction
			Gancao Decoction
			Dahuang Decoction
			Zhaihu Decoction
			Puhuang Decoction
			Zhizi Decoction
			Shengdihuang Decoction
			Shengma Decoction

		Miscellaneous women's treatments	Zhuru Decoction
			Banxia Houpo Decoction
			Sanggenbaipi Decoction
		Menstruation block	Qianhu Mudan Decoction
			Danggui Decoction
			Shengdihuang Decoction
			Chuanxiong Decoction
		Shaoxiaokebaxieshi	Baixianpi Decoction
		Various diseases of the pediatric population	Shengma Decoction
			Puhuang Decoction
		Small intestinal fever and mouth sores	Chaihu Zexie Decoction
		Solid heat in the large intestine, abdominal distension and inability to pass through	Shengjiang Xiechang Decoction
		Excessive cold in the upper regions	Huangqilizhong Decoction
		Dry heaves from the upper burner	Houpo Decoction
		Vomiting due to excessive heat in the lower regions	Zhiou RenshenDecoction
		Lower scorched earth and cold	Renshen Xuqi Decoction
		Canker Sore	Huangqi Zhuye Decoction
			Loulu Decoction
			Xiaozhuli Decoction
			Huangqi Fuling Decoction
			Zhizi Decoction
			Wuli Decoction
		Lactation	Lianqiao Decoction
		Detoxify	Congbaichi Decoction
			Gancao Decoction
			Guixin Decoction
			Renshen Decoction
			Shengmaimendong Decoction
			Dahuang Decoction
			Renshen Decoction
			Shengui Decoction Prescription
			Dahuang Huangqin Decoction
			Zhizichi Decoction

			Lizhong Decoction
			Renshen Decoction
			Binglang Decoction
		Sudden Death	Huanhun Decoction
		Injury such as Contusion	Jiaoai Decoction
			Dajiaoai Decoction
			Zhupi Decoction
			Taoren Decoction
**Classification of diseases**	**Types**		**Commonly Used Prescriptions**
Diseases of the Lungs	Cough	Cough of various qi	Houpo Decoction
		Palpitation	Jupi Decocton
		Lung Qi Deficiency	Bufei Decoction
		Lung Qi Deficiency,Lumbago	Xiaojianzhong Decoction
		Cough	Guiling Wuwei Gancao Decoction
			Linggan Wuwei Jiangxin Decoction
			Guiling Wuwei Gancao Quguijiajiangxinbanxia Decoction
			Jiajiangxinbanxia Xingren Decoction Prescription
			Qinganwuwei jiajiangxinbanxingdahuang Decoction
			Xiaoqinglong jia Shigao Decoction
			Shegan Mahuang Decoction
			Houpo Mahuang Decoction
			Maimendong Decoction
			Mahuang Shigao Decoction
			Shizao Decoction
			Wenpi Decoction
			Baibugen Decoction
			Haizao Decoction
			Baiqian Decoction
			Zhupi Decoction
	Lung Abscess	——	Jiegeng Decoction
			Xiefei Decoction
	Lung Consumption	Severe Lung Qi Deficiency	Mahuangyinqi Decoction
			Banxia Decoction
			Houpo Decoction
			Huangqi Decoction
			Daqianhu Decoction

			Zhuye Decoction
			Guizhiqushaoyao jiazaojia Decoction
	Children's Fetish Knot Distension and Fullness	——	Ziwan Decoction
			Wuweizi Decoction
			Shegan Decoction
			Guizhi Decoction
	Pulmonary Impotence	——	Gancao ganjiang Decoction
			Gancao Decoction
	Pediatric Typhoid Fever	——	Maimendong Decoction
			Shaoyaosiwujieji Decoction
			Mahuang Decoction
			Wuweizi Decoction
			Shengma Decoction
			Dahuang Decoction
			Zhuye Decoction
			Tiaozhong Decoction
			Shengdihuang Decoction
			Hengshan Decoction
	Cholera		Guizhi Decoction
			Guizhi plus gui Decoction
			Guizhi plus houpoxingzi Decoction
			Guizhi plus fuzi Decoction
			Guizhi reduce Shaoyao plus Fuzi Decoction
			Guizhi Gancao Longgu Muli Decoction
			Guizhi Renshen Decoction
			Guizhi Fuzi Decoction
			Guizhi reduce Gui plus Fulingbaizhu Decoction
			Guizhi plus Shaoyaoshengjiang geyiliang Renshensanliangxinjia Decoction
			Guizhi Mahuanggeban Decoction
			Mahuang Decoction
			Daqinglong Decoction
			Xiaoqinglong Decoction
			Guizhier Mahuangyi Decoction
			Guizhier Yuebiyi Decoction
			Mahunag Xixin Fuzi Decoction
			Mahuang Fuzi Gancao Decoction
			Mahuang Xingren Gancao Shigao

			Decoction
			Gegen Decoction
			Gegen plus Banxia Decoction
			Gegen Huangqin Huanglian Decoction
			Guizhi plus Gegen Decoction
			Baihu Decoction
			Baihu plus Renshen Decoction
			Tiaowei Chengqi Decoction
			Daxianxiong Decoction
			Xiaoxianxiong Decoction
			Zhizichi Decoction
			Zhizi Gancao Chi Decoction
			Zhizi Shengjiang Chi Decoction
			Zhizi Houpo Decoction
			Zhizi Ganjiang Decoction
			Zhizi Baipi Decoction
			Xiaochaihu Decoction
			Dachaihu Decoction
			Chaihu plus Mangxiao Decoction
			Chaihu Guizhi Decoction
			Chaihu Guizhi Ganjiang Decoction
			Huangqin Decoction
			Huangqin plus Banxia Shengjiang Decoction
			Dahuang Huanglian Xiexin Decoction
			Fuzi Xiexin Decoction
			Gancao Xiexin Decoction
			Banxia Xiexin Decoction
			Shengjiang Xiexin Decoction
			Ganjiang Huangqin Huanglian Renshen Decoction
			Huanglian Decoction
			Tongmai Sini plus Zhudanzhi Decoction
			Fuling Sini Decoction
			Fuzi Decoction
			Gancao Ganjiang Decoction
			Shaoyao Gancao Decoction
			Shaoyao Gancao Fuzi Decoction
			Zhigancao Decoction
			Chishizhi Yuyuliang Decoction

			Taohua Decoction
			Zhuling Decoction
			Mahuang Shengma Decoction
			Gancao Fuzi Decoction
			Baitouweng Decoction
			Guizhi Fuzi reduce gui plus Baizhu Decoction
			Banxia Houpo Decoction
			Gualou Xiebai Banxia Decoction
			Lingguizhugan Decoction
			Zhuli Decoction
Disease of the Heart	Tachycardia	Pregnancy Vexation(irritability and palpitations in mid-pregnancy)	Dabuxin Decoction
		Palpitations of Weak Heart Qi	Yuanzhi Decoction
		Shaking in Fear	Fushen Decoction
			Buxin Decoction
			Xiaodingxin Decoction
			Dadingxing Decoction
			Jingli Decoction
			Zhenxin Decoction
			Shigao Decoction
	Chest Impediment	Solid heat in the heart with boredom	Xiexin Decoction
		Constipation in the lower regions of the heart	Guixinsanwu Decoction
		Heart pain	Wuxin Decoction
			Gaoliangjiang Decoction
			Wenzhongdanggui Decoction
			Yangroudanggui Decoction
			Wenpi Decoction
			Gualou Decoction
		Chest Impediment	Zhishi Xiebai Guizhi Decoction
			Fuling Decoction
			Tongqi Decoction
			Qianhu Decoction
			Binglang Decoction
			Dahuangxiere Decoction
	Heart-Laore	——	Shengma Decoction
			Mahuangtiaoxinxiere Decoction

			Dahuanghuanglianxiexin Decoction
	Other Diseases	Heart Qi Deficiency, hematemesis	Zhuli Decoction
		Dreams, joy, laughter, fear, palpitations, restlessness.	Fulingbuxin Decoction
		Heart Qi Deficiency	Banxiabuxin Decoction
		Heart deficiency and cold, heart swelling and fullness	Fangfeng Decoction
Brain Disease	Dazzling	Dazzling	Dadouzi Decoction
	Stroke	Postpartum Stroke	Duhuozi Decoction
			Xiaoduhuo Decoction
			Gancao Decoction
			Duhuo Decoction
			Zhuye Decoction
			Fangfeng Decoction
			Duhuo Decoction
			Dadou Decoction
			Wushi Decoction
			Sishi Decoction
			Xiaochaihu Decoction
			Gancao Decoction
			Gegen Decoction
			Fushen Decoction
			Yuanzhi Decoction
			Fuling Decoction
			Anxin Decoction
			Xiaoxuming Decoction
		Stroke	Daxuming Decoction
			Xizhouxuming Decoction
			Paifeng Decoction
			Dabafeng Decoction
			Wutou Decoction
			Dafangfeng Decoction
			Chuanxiong Decoction
		Thief Wind Stroke	Yiyuanmahuangxuming Decoction
			Wenzhongshengjiang Decoction
			Wutou Decoction
			Fangfeng Decoction
			Qianghuo Decoction
			Fangji Decoction
			Dazao Decoction

			Fangfeng Decoction
		Contrary Wind	Gegen Decoction
			Zhongjingsanhuang Decoction
			Bailianyiyi Decoction
			Duhuojisheng Decoction
			Zhuli Decoction
		Post-stroke Hemiplegia	Duhuo Decoction
		Severe stroke coma	Canggongdanggui Decoction
		Opitoshtonous	Fushen Decoction
		Cardiac Diseases - Head and Face Wind	Xijiao Decoction
			Dahuang Decoction
	Epilepsy	Epilepsy	Zengsunxuming Decoction
			Shigao Decoction
			Banxia Fuling Decoction
Diseases of the spleen and stomach system	Vomiting	Obstruction of Pregnancy	Qingzhuru Decoction
			Jupi Decoction
			Dabanxia Decoction
		The stomach does not accept food and vomits when it is eaten	Banxia Decoction
		Vomiting	Qianhu Decoction
			Xiaomai Decoction
			Jupi Decoction
			Dahuanggancao Decoction
			Zhupi Decoction
	Choke	Choke	Ganjiang Decoction
			Tongqi Decoction
			Shaoyao Decoction
	Abdominal Pain	Postpartum Abdominal Pain	Danggui Decoction
			Yangrou Decoction
			Yangroudanggui Decoction
			Yangrouduzhong Decoction
			Yangroudihuang Decoction
			Neibudangguijianzhong Decoction
			Neibuchuanxiong Decoction
			Dabuzhongdanggui Decoction
			Wuzhuyu Decoction
			Puhuang Decoction
			Baijiang Decoction
			Duhuo Decoction
			Shaoyaohuangqi Decoction
			Baitouweng Decoction

	Loose Bowels	Postpartum Dysentery	Biejia Decoction
			Gandihuang Decoction
			Shengdihuang Decoction
			Sanhuangbaitouweng Decoction
	Typhoid	Dysentery characterized by heat	Baitouweng Decoction
			Fuling Decoction
			Wenpi Decoction
		Dysentery	Dataohua Decoction
			Cangmi Decoction
			Fuzi Decoction
			Duanli Decoction
			Xiexin Decoction
			Xiangsu Decoction
			Wenzhong Decoction
		Pediatric Dysentery	Wenzhong Dahuang Decoction
			Dahuang Decoction
			Sanhuang Decoction
	Insomnia	——	Dahuangxiere Decoction
	Other Diseases	Jaundice due to spleen fever	Banxia Decoction
		Depletion of the spleen	Buwei Decoction
		Bitterness in the mouth	Wenwei Decoction
		Bloating	Dabanxia Decoction
			Fuzi Jingmi Decoction
			Houposanwu Decoction
			Houpoqiwu Decoction
			Wuzhuyu Decoction
			Dagui Decoction
			Banxia Decoction
		Chronic cold and heat	Shengjiang Decoction
			Gancao Decoction
			Zhuyu Xiaoshi Decoction
			Dahuang Fuzi Decoction
			Dawutou Decoction
			Wushi Decoction
			Zhuye Decoction
			Banxia Decoction
			Chengqi Decoction
			Gandihuang Decoction
disease in the Liver and gallbladder system	Duress	Post-partum dystocia	Bugan Decoction
		Liver qi deficiency, dystocia	Binglang Decoction
		Liver deficiency and	Xianxiong Decoction

		cold, distension and dystocia	
	Aggregation	Accumulation of hardened mucus	Gansui Decoction
			Loulu Decoction
	Other Diseases	Pediatric carbuncle and scrofula	Mahuang Decoction
			Jupitongqi Decoction
		Cramps	Liyu Decoction
Renal Diseases	Edema	Edema in pregnancy, dysuria	Gualou Decoction
	Other diseases	Postnatal Gonorrhea	Zhuye Decoction
			Xieshen Decoction
		Abdominal distension and fullness with solid heat in the kidneys	Zhizi Decoction
		Excess heat in the kidneys caused by internal heat	Zhuyehuangqin Decoction
		Essential qi Deficiency	Sanhuang Decoction
		Guji	Shenzhuo Decoction
		Lumbago	Shengdihuang Decoction
Diseases of Qi,Blood,Fluid and Humor	Hematemesis	——	Jianzhong Decoction
			Baiye Decoction
			Zelan Decoction
			Xijiaodihuang Decoction
			Danggui Decoction
			Zhuru Decoction
			Maimendong Decoction
			Xiaobanxia Decoction
	Phlegm-retained Fluid	——	Gancao Decoction
			Mufangji Decoction
			Houpo Dahuang Decoction
			Xiaobanxia plus Fuling Decoction
			Gansuibanxia Decoction
			Daguling Decoction
			Fuling Decoction
			Dabanxia Decoction
			Banxia Decoction
			Danggui Decoction
			Wuzhuyu Decoction
			Qianhu Decoction
			Xuanfuhua Decoction
			Jiangjiao Decoction
			Duange Decoction

			Yangrou Decoction
	Consumptive Disease	Postpartum Consumptive Disease	Zhushen Decoction
			Yaorouhuangqi Decoction
			Dangguishaoyao Decoction
			Xingren Decoction
			Wushi Decoction
			Sanshi Decoction
			Neibu Huangqi Decoction
			Wuzhuyu Decoction
			Liyu Decoction
			Guizhi plus Fuzi Decoction
			Xiebai Decoction
		Postpartum Vexation of Deficiency Type	Renshen Danggui Decoction
			Ganzhuru Decoction
			Zhimu Decoction
			Zhuye Decoction
			Danzhuru Decoction
			Shaoyao Decoction
			Wendan Decoction
		Gallbladder Qi Deficiency leads to Vexation of Deficiency Type	Suanzao Decoction
			Zhizi Decoction
			Jianzhong Decoction
		Consumptive Disease	Xiaojianzhong Decoction
			Huangqinjianzhong Decoction
			Qianhujianzhong Decoction
			Lelingjianzhong Decoction
			Huangqin Decoction
			Dajianzhong Decoction
			Shenli Decoction
			Zengsunshenli Decoction
			Ningtuo Decoction
			Renshen Decoction
			Diyizhuli Decoction
Disease in the limbs and meridians	Paralysis	Common cold sore	Disanzhuli Decoction
			Mahuang Decoction
			Dierfuduhuo Decoction
			Disifufuhuo Decoction Prescription
			Duhuo Decoction
			Yuebi Decoction
			Fengyin Decoction
			XIjiao Xuanfuhua DecoctionPrescription

			Daxijiao Decoction
			Wuzhuyu Decoction
			Xiaofengyin Decoction
			Siwu Fuzi Decoction
			Banxia Decoction
			Zisuzi Decoction
			Fuzi Decoction
			Fangfeng Decoction
			Gancao Decoction
			Jiefengbi Decoction
	Atrophy	——	Xizhouxuming Decoction
			Xiegan Decoction
Diseases Of Seven Orifices	Eye disease	Be Unable to See Clearly	Shengdihuang Decoction
	Nose disease	Nosebleed	Piaoxiaodangbao Decoction
Other diseases	——	Sterility	Wuciji Decoction Prescription
		Prevent Abortion	Butai Decoction
			Aiye Decoction Prescription
			Huanglian Decoction
			Xiongji Decoction Prescription
			Fushen Decoction
			Juhua Decoction Prescription
			Anzhong Decoction Prescription
			Maimendong Decoction Prescription
			Chaihu Decoction
			Congbai Decoction Prescription
			Xingren Decoction
			Shaoyao Decoction
			Banxia Decoction
			Zhushen Prescription
			Congbai Decoction
		Threatened Abortion	Xuanfuhua Decoction
			Jiaoai Decoction
			Danxingguijian Decoction
		Promote lactation	Gandihuang Decoction
		Lochia	Zelan Decoction
			Gancao Decoction
			Dahuang Decoction
			Zhaihu Decoction
			Puhuang Decoction
			Zhizi Decoction
			Shengdihuang Decoction

			Shengma Decoction
			Zhuru Decoction
		Miscellaneous women's treatments	Banxia Houpo Decoction
			Sanggenbaipi Decoction
			Qianhu Mudan Decoction
		Menstruation block	Danggui Decoction
			Shengdihuang Decoction
			Chuanxiong Decoction
			Baixianpi Decoction
		Shaoxiaokebaxieshi	Shengma Decoction
		Various diseases of the pediatric population	Puhuang Decoction
			Chaihu Zexie Decoction
		Small intestinal fever and mouth sores	Shengjiang Xiechang Decoction
		Solid heat in the large intestine, abdominal distension and inability to pass through	Huangqilizhong Decoction
		Excessive cold in the upper regions	Houpo Decoction
		Dry heaves from the upper burner	Zhiou RenshenDecoction
		Vomiting due to excessive heat in the lower regions	Renshen Xuqi Decoction
		Lower scorched earth and cold	Huangqi Zhuye Decoction
		Canker Sore	Loulu Decoction
			Xiaozhuli Decoction
			Huangqi Fuling Decoction
			Zhizi Decoction
			Wuli Decoction
			Lianqiao Decoction
		Lactation	Congbaichi Decoction
		Detoxify	Gancao Decoction
			Guixin Decoction
			Renshen Decoction
			Shengmaimendong Decoction
			Dahuang Decoction
			Renshen Decoction
			Shengui Decoction Prescription
			Dahuang Huangqin Decoction
			Zhizichi Decoction

			Lizhong Decoction
			Renshen Decoction
			Binglang Decoction
			Huanhun Decoction
		Sudden Death	Jiaoai Decoction
		Injury such as Contusion	Dajiaoai Decoction
			Zhupi Decoction
			Taoren Decoction

C.2 Representative Example: Guidelines for the Administration of Gui Zhi Decoction

C.2.1 Basic Effects

Gui Zhi Dcoction functions in relieving muscle and publishing, harmonizing Ying and Wei. It is used for treating external wind-cold syndrome. Malignant wind and fever, sweating and headache, nasal sounding and dry vomiting, white moss and no thirst, floating or weak pulse.

C.2.2 General Guidance

C.2.2.1 Taking Time

To be taken on an empty stomach.

C.2.2.2 Frequency of Dosage

Flexibly control the number of times Gui Zhi Decoction is to be taken according to changes in condition, 1-3 times a day.

C.2.2.3 Taking Temperature

37℃～40℃ is suitable.

C.2.2.4 Dosage

Adults should take 200mL per dose. Elderly people, children, pregnant women and nursing mothers should follow the doctor's instructions.

C.2.2.5 Course of Treatment

The duration of Gui Zhi Decoction is related to the change of condition. If the condition improves after one dose of the medicine with sweating, the medicine can be stopped; if there is no sweating, continue to take the medicine; if there is no sweating, take all 3 doses in 1 day; if the patient's condition is serious and the change of condition is closely watched during the taking of the medicine, the duration of the medicine can be prolonged relatively; if no sweating occurs all the time, the medicine has to be taken for 2-3 consecutive days, or follow the doctor's instruction.

C.2.2.6 Storage Guidelines

Vacuum-sealed packages of Chinese medicine decoctions should be stored in the refrigerator within 2-8℃ for a period of two weeks; if there are abnormal phenomena such as bulging of the bag of medicinal liquid or discoloration, change of taste or bubbling of the medicinal liquid in the process of taking the Chinese medicine decoctions, it is not suitable to take the Chinese medicine decoctions.

C.2.3 Personalized Guidance

C.2.3.1 Contraindications to Use

C.2.3.1.1 Drug-drug

Gui Zhi Decoction contains licorice, at the same time of taking this formula, it is not allowed to take seaweed, halberd, coriander, Gan Sui drinking tablets or proprietary Chinese medicines or formulated preparations containing its drinking tablets; when it is used with cardiac glycoside drugs digoxin and digitalis, pay attention to whether there is any digoxin intoxication reaction occurring; and blood pressure-lowering drugs lisdexamfetamine, glucose-lowering drugs, acidic western medicines, enzyme preparations, antimicrobials erythromycin, tetracycline, chloramphenicol, warfarin, coumarin anticoagulant drugs, Salicylic acid derivatives of drugs, potassium-excluding diuretics, carbamazepine, methyldopa, H2 receptor blockers, vitamin A and other drugs used together, may reduce its efficacy; and with tricyclic antidepressants used together, may make the tricyclic antidepressant metabolites increase, triggering adverse reactions.

Patients should not add sugar in the process of taking Gui Zhi Decoction.

If there is scorched decocted of the decoction, it should not be taken.

C.2.3.1.2 Drug-Population

Gui Zhi is pungent, warm and helps heat, warms the meridians, pregnant women and those with excessive menstruation should take it under the guidance of a doctor.

Ginger helps fire and harms yin. People with heatiness and heart deficiency should not take Gui Zhi Decoction.

Patients with accumulation of food, accumulation of worms, caries and pain, phlegm-heat cough should not take Gui Zhi Decoction.

Patients who have been drinking alcohol for a long time or are alcoholics should not take Gui Zhi Decoction.

C.2.3.1.3 Drug-Disease

Gui Zhi is pungent, warm and helps heat, easily injuring yin and moving Blood. It is not recommended to take Gui Zhi Decoction in cases of externally sensed fever, yin deficiency and fire, Blood heat, dampness and fullness in the epigastrium and abdomen.

Gui Zhi Decoction is contraindicated in cases of external wind-cold with solid surface and no sweating.

C.2.3.1.4 Drug-Food/Health Supplements

Cold, raw, sticky and slippery substances, meat and noodles, five pungent substances, wine and cheese, and foul smells should be prohibited while taking Gui Zhi Decoction.

C.2.3.1.5 Drug-Environment

Caution should be taken to avoid wind while taking Gui Zhi Decoction.

C.2.3.2 Post-Medication Care

After taking Gui Zhi Decoction, sip more than one liter of hot thin porridge. It is better if the body is hot and slightly sweaty.

C.2.3.3 Effects on Excretory Production After Drug Administration

Observe the state of sweating, and do not make it as if water is flowing.

C.2.3.4 Medication Monitoring

Monitor changes in condition and degree of sweating.

References

[1] World Federation of Societies of TCM.SCM 0008-2011 International Standard for Grading Professional and Technical Titles of TCM Practitioners, People's Health Publishing House, 2011.

[2] FIP GUIDELINES FOR THE LABELS OF PRESCRIBED MEDICINE, 2001, Singapore.

[3] SCM 0055-2020 Requirements for prescribing, dispensing, administering and decoction of Chinese medicine decoctions Part 4: Requirements for decoction of Chinese medicine decoctions.

[4] T/CACM 1370.1-2021 Technical Specification for Revision of Safety Information Program for Listed Proprietary Chinese Medicines General Principles

[5] ZHAI Huaqiang, HUANG Hui, ZHENG Huzhan. Practical techniques of clinical preparation of traditional Chinese medicine [M]. Beijing: People's Health Publishing House. 2011. 8.

[6] Liu QH, Tong SL. Study on the method of decocting soup [M]. Beijing:Science Press. 2016. 6.

[7] Li GH. Translation of the classic Typhoid Fever for modern physicians [M]. Beijing:Traditional Chinese Medicine Ancient Books Publishing House. 2013. 6.

[8] Wang B, Wu JK. The illustrated thousand gold formula [M]. Jiangsu: Jiangsu Phoenix Science and Technology Press. 2020. 1.

[9] National Pharmacopoeia Commission of China. Pharmacopoeia of the People's Republic of China (2020 Edition, Part I) [M]. Beijing:China Pharmaceutical Science and Technology Press. 2020. 1.

[10] Li Ji, Zuo Zhengyun. Formulary ("14th Five-Year Plan" Textbook) [M]. Beijing: China TCM Press, 2021.

[11] Li Qingye, Gao Lin, Wang Yunge, Zhao Hui, Yang Zhen. Tangtou Geji in vernacular language (6th ed.) [M]. Beijing:People's Health Publishing House. 2016.

[12] Li Peisheng. Lecture notes on typhoid fever (for Chinese medicine specialty) [M]. Shanghai:Shanghai Science and Technology Press, 2017. 4.

[13] Editorial Committee of National Guidelines for Clinical Application of Essential Drugs. Guide to clinical application of national essential drugs [M]. Beijing:People's Health Publishing House, 2011.

[14] Zhang Bing, Zhou Zhenxiang. Clinical pharmacotherapy of traditional Chinese medicine [M]. Beijing: People's Health Press, 2016.

[15] Cao JL, Zhen HS. Interaction between proprietary Chinese medicines and western medicines [M]. Beijing:People's Health Press, 2016.

[16] Wu Mianhua, Shi Yan. Internal medicine of traditional Chinese medicine [M].

Beijing:China TCM Press, 2021.
[17] Zhang Bing. Pharmacovigilance in Chinese medicine [M]. Beijing:People's Health Publishing House, 2015.
[18] Yihua. Shelf life of traditional Chinese medicine [J]. Jiangsu Health Care, 2021(06): 44.
[19] Zheng Hong, Xu Hongwei, Qin Jinghai, et al. Influence of different treatment methods on the shelf-life of traditional Chinese medicine decoctions in soup packers[J]. Research on Traditional Chinese Medicine, 2014, 27(03): 70-72.
[20] ZHANG Lishuang, JIANG Feng, JIN Xinyao, et al. Literature-based analysis of the method of taking traditional Chinese medicine decoctions[J]. Global TCM, 2018, 11(12): 1946-1949.
[21] Zhai HQ, Wang YP. Commonly used clinical formulas and proprietary Chinese medicines [M]. Beijing:People's Health Publishing House, 2020.
[22] Li YF, Zhang SS, Guo RJ, Zhai HQ. Rational combination prescription of drugs for internal diseases [M]. Zhengzhou: Henan Science and Technology Press, 2021.
