# Supplementary material for: A new evaluation methodology study - Integrating ‘ancient literature - clinical research - expert consensus’ firstly proposes eight elements for taking Chinese medicine decoctions
Source: Front Pharmacol. 2025 Aug 8;16:1585428. doi: 10.3389/fphar.2025.1585428 (PMC12371335; doi:10.3389/fphar.2025.1585428)
Supplement: Supplementary file 3 [file DataSheet1.docx]

Supplementary Material

# Supplementary material

Awareness and Demand Research for Taking Commonly-Used Chinese Medicine Decoction

Dear Medical Workers:
      In order to strengthen the construction of Chinese medicine clinical pharmacist team, standardize the normality of Chinese pharmacists in the process of medication instruction of Chinese medicine decoctions, and improve the level of Chinese medicine clinical pharmacy services, this questionnaire is designed to understand the current service status and service demand of medication instruction of commonly used Chinese medicine decoctions in China, so as to promote the safety and efficacy of Chinese medicine decoctions in the process of clinical use. The questionnaire consists of 32 questions, covering three parts: basic information, awareness of medication instruction of Chinese medicine decoctions and medication demand.
       The questionnaire is expected to take up about 5 minutes of your time and involves four types of questions:
       1. Multiple-choice questions - please select the appropriate option according to the question;
       2. Fill-in-the-blank questions - please fill in your true thoughts directly in the blank position;
       3. Sorting questions - please follow the question prompts to select the appropriate option and sort the order of choice;
       4. Scoring questions - please rate the importance of the questions according to the prompts.
      We promise that this questionnaire will not disclose your personal information and will be answered anonymously. Please answer the questionnaire carefully, as your answers and suggestions are crucial to the research of the topic, and we thank you for your support and cooperation!

--Standardization Research Center of Traditional Chinese Medicine Dispensing

Beijing University of Chinese Medicine

**Part I Basic Information**

1. Your work area is [Fill in the Blank] Please directly fill in the name of the city where your work unit is located, such as: Beijing _________________________________

2. The nature of your work unit is [Single Choice]

| ○Chinese Medicine Hospital |
| --- |
| ○Western Medicine Hospital |
| ○Chinese and Western medicine hospital |
| ○Community Hospital |
| ○Other _________________ |

3. The type of your workplace is [Single Choice]

| ○Grade IIIA hospital |
| --- |
| ○Grade III hospital |
| ○Grade II hospital |
| ○Grade I hospital |
| ○Other _________________ |

4. What is your position in your work unit?[Single Choice]

| ○Physician |
| --- |
| ○Pharmacist |
| ○Nurse |
| ○Other _________________ |

5. Your work department is [Fill in the Blank] Please fill in your work department directly, such as: Department of Traditional Chinese Medicine _________________________________

relies on the first option of Q4

6. Your years of working experience is [Single Choice]

| ○5 years and below |
| --- |
| ○6-10 years |
| ○11-20 years |
| ○21 years and above |

7. Your job position in the work unit is [Single Choice]

| ○Chinese medicine clinical pharmacist |
| --- |
| ○Western medicine clinical pharmacist |
| ○Other _________________ |

Depends on the 2nd option of Q4

8. Your highest level of education is [Single Choice]

| ○ Bachelor's degree or below |
| --- |
| ○Master's degree |
| ○Doctoral degree or above |

**Part II Survey on Awareness of the Guidelines for Taking Commonly Used Chinese Medicine Decoctions**

9. In your medical organization, the frequency of prescribing Chinese medicines decoctions is [Single Choice]

| ○ Very frequently |
| --- |
| ○ Frequently |
| ○ Occasionally |
| ○ Hardly ever |

10. The probability of prescribing Chinese medicine decoctions in combination with other dosage forms or combining Chinese and Western medicines is [Single Choice]

| ○Very frequently |
| --- |
| ○Frequently |
| ○ Occasionally in combination with other treatments |
| ○Almost never in combination with other drugs |

Depends on the 1st;2nd;3rd option in Q9

11. Does the pharmacy service carried out by your medical organization include guidance on taking Chinese medicine decoctions [Single Choice]

| ○Yes |
| --- |
| ○No |
| ○Unknown |

12. Who is mainly responsible for providing guidance on taking Chinese medicine decoctions in your medical organization?[Multiple Choice]

| □Physician |
| --- |
| □Pharmacist |
| □Nurse |
| □Other _________________ |

13. Have you ever instructed patients on taking Chinese medicine decoctions? [Single Choice]

| ○Yes |
| --- |
| ○No |

14. Sorting question: In the process of giving instructions on taking Chinese medicine decoctions, what are the main aspects that you will give instructions on [Sorting Question, please fill in the numbers in the middle bracket in order] Please select the contents of the instructions on taking medication that you are likely to give in your actual work based on your work experience and sort them in descending order by considering the frequency of the instructions on taking medication and frequency of the patients' counseling.

| [ ]Basic therapeutic effects of Chinese medicine decoctions |
| --- |
| [ ]Toxic side effects of Chinese medicine decoctions |
| [ ]Expected time of onset of effect after taking Chinese medicine decoctions and measures to cope with the lack of onset of effect |
| [ ]The time, temperature, dosage, method, frequency and duration of taking Chinese medicine decoctions |
| [ ] Symptoms, signs and tests that should be monitored during administration, and explanation of the possible effects of the Chinese medicine decoctions on clinical tests or changes in the color of excreta, etc. |
| [ ] Common or serious adverse reactions that may occur, preventive measures that can be taken against adverse reactions, and treatment measures after the occurrence of adverse reactions |
| [ ]The possible consequences of medication errors (e.g., omission or overdose) and the measures to deal with them. |
| [ ]Potential drug-drug, drug-food/nutraceutical, drug-disease and drug-environment interactions or contraindications |
| [ ]Suitable storage conditions for Chinese medicine decoctions and the circumstances under which they cannot be taken further (e.g. swelling of the bag of Chinese medicine decoctions, etc.) |
| [ ]How to keep a record of medication administration and self-monitoring, and how to contact the pharmacist |
| [ ]Other |

Depends on the 1st option of Q13

15. In the process of guiding the administration of Chinese medicine decoctions, which of the following groups of people is your main target of guidance [Matrix Text Question] [Enter a number from 0 to 100] Please rate the importance of the target of your guidance based on your actual clinical work experience, with 85-100 as close attention, 75-84 as relative attention, 60-74 as occasional attention, and 59 points and below as almost No concern

| Ordinary patients | ________________________ |
| --- | --- |
| Patients in special groups (the elderly, children, liver and kidney insufficiency, having a plan to prepare for pregnancy, pregnant or lactating women, etc.) | ________________________ |
| Allergic patients | ________________________ |
| Patients taking Chinese medicine decoctions for the first time | ________________________ |
| Patients with poor literacy or understanding | ________________________ |

Depends on the 1st option of Q13

16. In the process of giving instructions to patients on taking medicines, how often do you give instructions on the temperature of taking Chinese medicine decoctions [Single Choice]

| ○Very frequently |
| --- |
| ○Only give instructions on decoctions with special temperatures |
| ○Only respond to patient inquiries |
| ○Never give instructions on the temperatures of medication |

Depends on the 4th option of Q14

17. How often do you give instructions on the time of taking Chinese medicine decoctions to patients [Single Choice]

| ○Very frequently |
| --- |
| ○Only give instructions on decoctions with special times |
| ○Only respond to patient inquiries |
| ○Never give instructions on the time of medication |

Depends on the 4th option of Q14

18. In your opinion, the best time to take decoctions with nourishing effects is [Multiple Choice]

| □Before meals |
| --- |
| □On an empty stomach |
| □After meal |
| □Before bedtime |
| □Irregularly |
| □Other _________________ |

Depends on the 1st;2nd;3rd option of Q17

19. In your opinion, the best time to take decoctions for treating diseases of the spleen, stomach and gastrointestinal area is [Multiple Choice]

| □Before meals |
| --- |
| □On an empty stomach |
| □After meal |
| □Before bedtime |
| □Irregularly |
| □Other _________________ |

Depends on Q17, option 1;2;3

20. During the process of instructing patients on taking decoctions, how often do you instruct on post-drug care [Single Choice]

| ○Very frequently |
| --- |
| ○Only give instructions on decoctions that require special care |
| ○Only respond to patients inquiries |
| ○Almost never give instructions on post-drug care |

Depends on Q14, options 1;3;5;6;7

21. How much importance do you attach to the possible adverse reactions and their preventive measures after taking Chinese medicine decoctions in the process of giving instructions on taking medicines? [Single Choice]

| ○Very important |
| --- |
| ○Only some of the decoctions will be emphasized on adverse reactions and relief measures |
| ○Chinese medicine decoctions have no toxic side effects and there is no need to consider adverse reactions in the course of taking the medicine |

Depends on options 1;2;3;6;7;9 in Q14

22. Under which of the following circumstances would you attach importance to the adverse effects of Chinese medicine decoctions [Matrix scale questions] Please choose according to your actual clinical experience in the use of medicines, and choose according to the degree of importance attached to it, and if you choose the other options in the "Other" entry except for the option of "Does not meet", please tick the appropriate box. If you choose any of the "Other" entries other than the "Noncompliance" option, please fill in the corresponding concerns after the corresponding scores; the rest of the entries do not need to be filled in.

|  | Not conforming | Somewhat conforming | Conforms | Relatively comforming | Quite conforming |
| --- | --- | --- | --- | --- | --- |
| Patients were informed of possible adverse reactions for all decoctions | ○ | ○ | ○ | ○ | ○ |
| Traditional Chinese medicine decoctions containing toxic herbal decoction pieces | ○ | ○ | ○ | ○ | ○ |
| Containing a large number of potent Chinese herbal decoction pieces | ○ | ○ | ○ | ○ | ○ |
| Special attention is paid to the use of special populations such as the elderly and children | ○ | ○ | ○ | ○ | ○ |
| Exceeding the clinically prescribed dosage or indications | ○ | ○ | ○ | ○ | ○ |
| Others | ○ | ○ | ○ | ○ | ○ |

Depends on the first two choices in Q21.

23. At present, in your medical organization, the main form of guidance on taking Chinese medicine decoctions is [Single Choice]

| ○ Verbal instruction during the process of patient's medication collection |
| --- |
| ○ Formation of a medication instruction sheet for patients' prescriptions, which is distributed to patients together with medications |
| ○ Combination of verbal instruction and medication instruction sheet |
| ○Other _________________ |

24. Do you think that appropriate guidelines for taking Chinese medicine decoctions are helpful in improving patients' medication adherence? [Single Choice]

| ○Yes |
| --- |
| ○No _________________ |
| ○Unknown |

25. Do you think appropriate guidnce on taking Chinese medicine decoctions is helpful in improving patients' rational use of decoctions?[Single Choice]

| ○Yes |
| --- |
| ○No _________________ |
| ○Unknown |

26. Sorting question: In the process of providing guidance on taking Chinese medicine decoctions in clinical practice, what are the main contents on which you base your guidance on taking Chinese medicine decoctions [Sorting Question, please fill in the numbers in parentheses] *Select your references and sort them according to the degree of importance of the references.

| [ ]Actual clinical experience in the use of medication |
| --- |
| [ ]Chinese medicine treatment guidelines for related diseases |
| [ ]Pharmacopoeia of the People's Republic of China |
| [ ]Specialized books such as "Formulary" and "Traditional Chinese Medicine Therapeutics". |
| [ ]Results of randomized clinical trials |
| [ ]Literature published in domestic and international journal databases |
| [ ]Classical books such as *Taiping Huimin Hejiaobingfang* |
| [ ]Others |

Depends on the 1st option of Q13

**Part III Survey on the Demand for taking commonly-used Chinese Medicine Decoctions**

27. Have you ever encountered any problems in giving instructions on taking Chinese medicine decoctions?

| ○ Yes |
| --- |
| ○ No |

Depends on the 1st option of Q13

28. What are the main confusions you have encountered when instructing patients on how to take Chinese medicine decoctions [Multiple Choice]

| □Don't know how to give instructions on this kind of decoctions |
| --- |
| □Don't know the source of the instructions on the use of the relevant decoctions |
| □No standardized medication instructions exist and the focus of the instructions is not clear |
| □Do not provide adequate answers to questions asked by patients |
| □There is confusion in remembering the precautions for the relevant decoctions |
| □Contradiction between what you know and what your physician has told you |
| □Other _________________* |

Depends on the 1st option of Q27

29. Sorting question: In your opinion, the main reason for the problems in the instruction of taking Chinese medicine decoctions is [Sorting Question, please put the numbers in brackets] *Please select the main reason you think for the problems in the instruction process, and sort them in descending order of importance.

| [ ] Lack of knowledge in related disciplines such as traditional Chinese medicine and pharmacognosy |
| --- |
| [ ]Lack of time and experience in the process of medication counseling due to large number of patients and heavy workload |
| [ ]Lack of standardization and process of medication instruction for Chinese medicine decoctions |
| [ ]The current model of medication guidance is rigid, resulting in a focus on medication guidance that does not meet individualized needs |
| [ ]Patients' distrust or lack of cooperation with pharmacists |
| [ ]Problems in communication between patients and pharmacists due to the limitations of patients' literacy level and other factors, which make it difficult for patients to understand some terminology |
| [ ]Other |

Depends on the 1st option of Q27

30. Do you think it is necessary to set up guidelines for taking Chinese medicine decoctions in clinical practice [Single Choice]

| ○Very necessary |
| --- |
| ○Necessary |
| ○Not necessary |
| ○Not necessary _________________ |

31. If you were to prepare a "Guidelines for taking commonly-used Chinese medicine decoctions", which of the following information would you like to see included in it [Matrix text question] [Enter a number from 0 to 100] *Please consider the importance of the following frameworks as well as your needs and rate them, with 90-100 as very important, 80-89 as important, 70-79 as important, 60-69 as important, 60-69 as important, 60-69 as important, and 60-69 as important. -79 is important, 60-69 is less important, and 59 and below is not important.

| Basic description |  |
| --- | --- |
| Names of Chinese medicine decoctions and their basic therapeutic effects | ______________________ |
| Estimated time of onset of action of Chinese medicine decoctions and countermeasures in case of failure to take effect | ______________________ |
| Time of taking Chinese medicine decoctions | ______________________ |
| Temperature of taking Chinese medicine decoctions | ______________________ |
| Frequency of taking Chinese medicine decoctions | ______________________ |
| Dosage of Chinese medicine decoctions | ______________________ |
| Methods of taking Chinese medicine decoctions | ______________________ |
| The course of treatment for taking Chinese medicine decoctions | ______________________ |
| Symptoms or tests that need to be monitored during the use of the Chinese medicine decoctions | ______________________ |
| Possible effects of Chinese medicine decoctions on clinical tests or changes in the color of excreta, etc. | ______________________ |
| Special instructions |  |
| Toxic side effects of Chinese medicine decoctions | ______________________ |
| Possible adverse reactions to Chinese medicine decoctions | ______________________ |
| Preventive and curative measures against the occurrence of adverse reactions | ______________________ |
| Possible outcomes or treatments in the event of medication errors | ______________________ |
| Potential drug-drug, drug-diet/nutraceutical, drug-disease, and drug-environment interactions or contraindications | ______________________ |
| Suitable storage conditions forChinese medicine decoctions and the conditions under which they should not be taken any further | ______________________ |
| How to keep a medication record and self-monitoring | ______________________ |
| How to contact a pharmacist | ______________________ |

32. If you have any other suggestions or comments on the preparation of the Guidelines for taking commonly-used Chinese medicine decoctions, please leave your valuable views here [Fill in the Blank]

________________________________

# Supplementary Figures and Tables

## Supplementary Figures


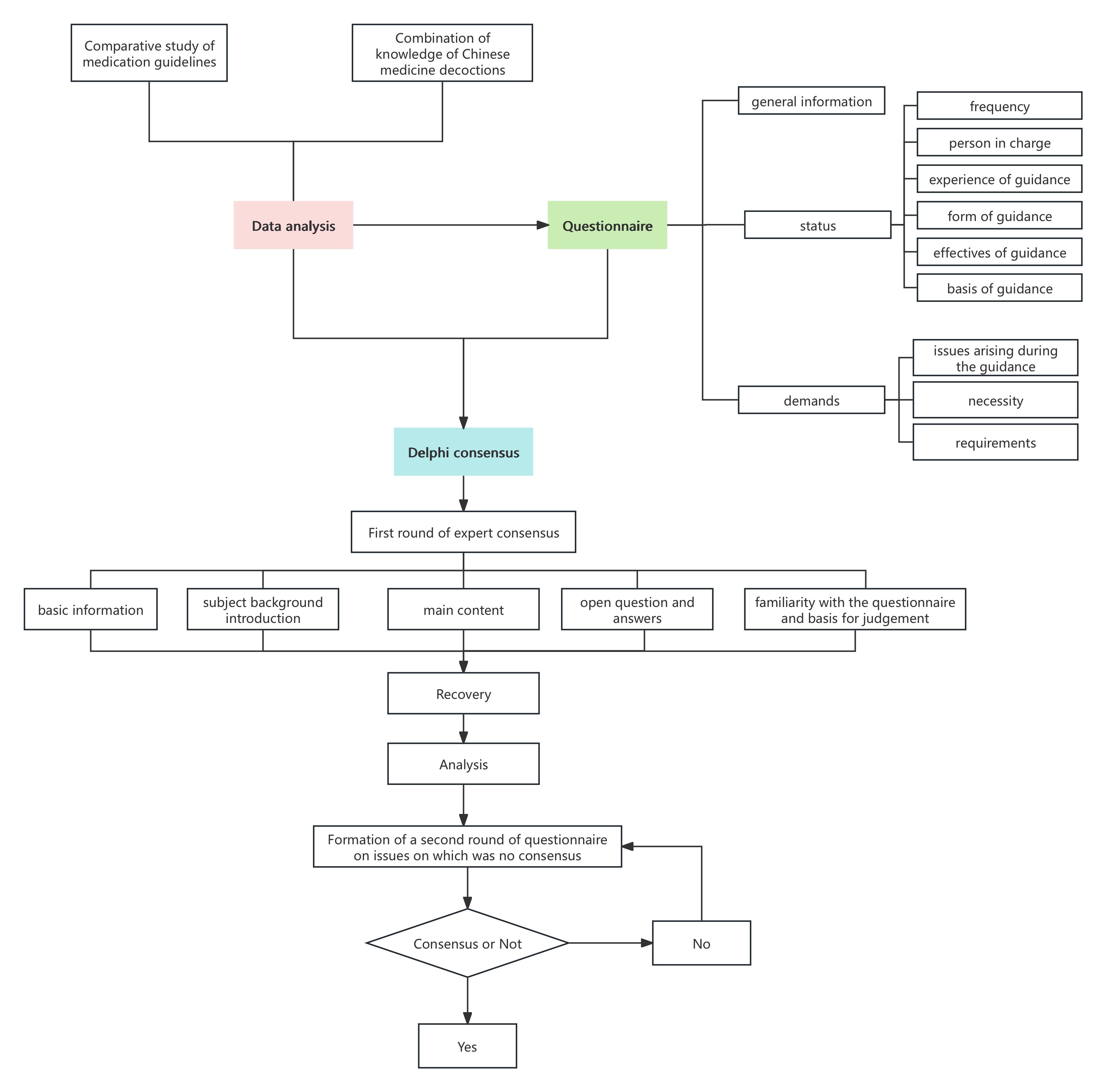


**Supplementary Figure 1.** Screening process for key elements of the medication guidelines


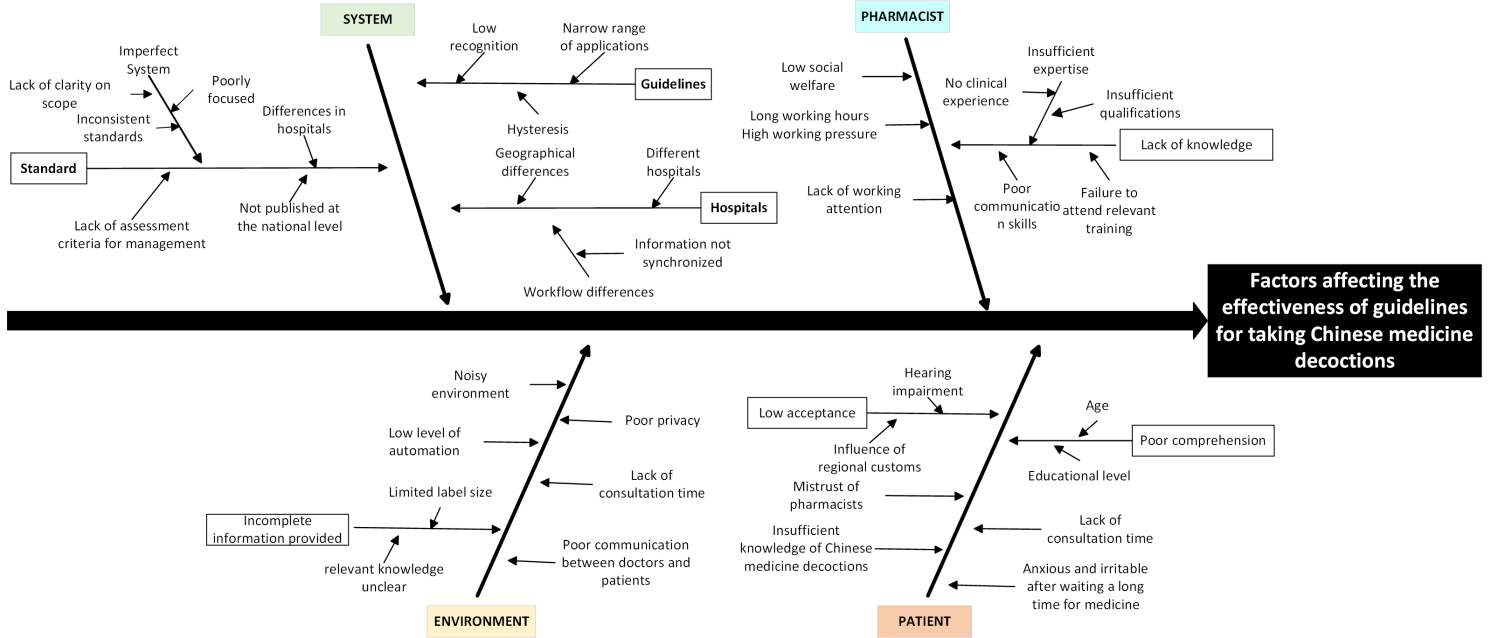


**Supplementary Figure 2.** Factors affecting the effectiveness of guidelines for taking Chinese medicine decoctions


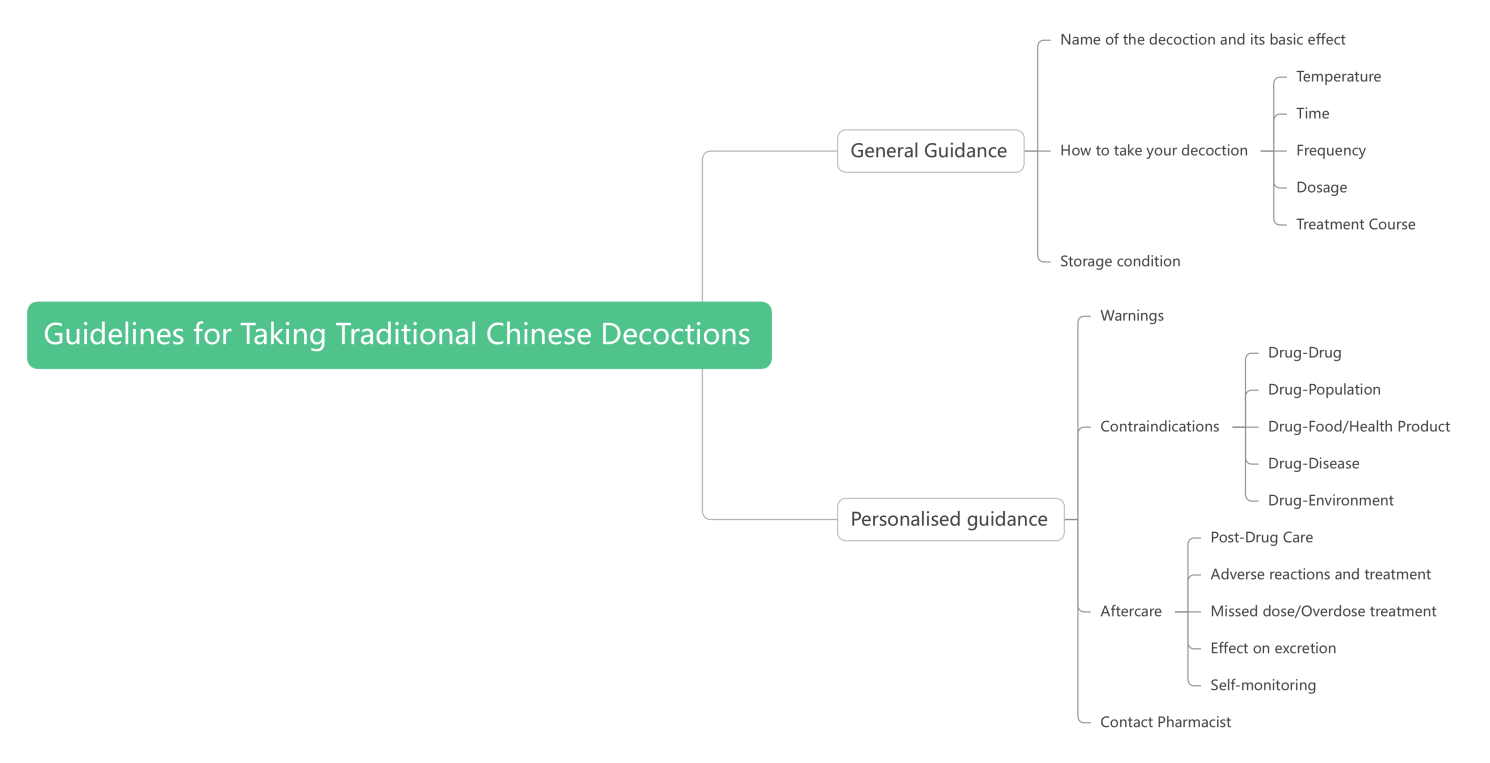


**Supplementary Figure 3.** Key elements affecting the efficacy of taking Chinese medicine decoctions
